# Serotonin Drives Predatory Feeding Behavior via Synchronous Feeding Rhythms in the Nematode *Pristionchus pacificus*

**DOI:** 10.1534/g3.117.300263

**Published:** 2017-09-12

**Authors:** Misako Okumura, Martin Wilecki, Ralf J. Sommer

**Affiliations:** Department for Evolutionary Biology, Max-Planck Institute for Developmental Biology, 72076 Tuebingen, Baden-Wuerttemburg, Germany

**Keywords:** predatory feeding behavior, serotonin, *tph-1*, *bas-1*, *Pristionchus pacificus*

## Abstract

Feeding behaviors in a wide range of animals are regulated by the neurotransmitter serotonin, although the exact neural circuits and associated mechanism are often unknown. The nematode *Pristionchus pacificus* can kill other nematodes by opening prey cuticles with movable teeth. Previous studies showed that exogenous serotonin treatment induces a predatory-like tooth movement and slower pharyngeal pumping in the absence of prey; however, physiological functions of serotonin during predation and other behaviors in *P. pacificus* remained completely unknown. Here, we investigate the roles of serotonin by generating mutations in *Ppa-tph-1* and *Ppa-bas-1*, two key serotonin biosynthesis enzymes, and by genetic ablation of pharynx-associated serotonergic neurons. Mutations in *Ppa-tph-1* reduced the pharyngeal pumping rate during bacterial feeding compared with wild-type. Moreover, the loss of serotonin or a subset of serotonergic neurons decreased the success of predation, but did not abolish the predatory feeding behavior completely. Detailed analysis using a high-speed camera revealed that the elimination of serotonin or the serotonergic neurons disrupted the timing and coordination of predatory tooth movement and pharyngeal pumping. This loss of synchrony significantly reduced the efficiency of successful predation events. These results suggest that serotonin has a conserved role in bacterial feeding and in addition drives the feeding rhythm of predatory behavior in *Pristionchus*.

Animals utilize diverse food sources and exhibit many different feeding behaviors depending on their ecology and physiology. In nematodes for example, their extreme habitat diversity is reflected by a range of different feeding adaptations and food sources including bacteria, fungi, protists, and plant roots (Yeates *et al.* 1993). While the neural mechanisms regulating bacterial feeding have been studied intensively in *Caenorhabditis elegans* (Avery 2012), the neural regulation of the feeding behaviors utilized by other diverse nematodes are unknown. One of the most striking examples of nematode behaviors is predatory feeding in which certain nematode species, such as *Pristionchus pacificus*, can use other nematodes as prey (Bento *et al.* 2010). Studying the different feeding strategies of these two species can therefore provide insights into the evolution of the neural mechanisms associated with feeding type switches and predation.

*P. pacificus* has been established as a satellite model organism for comparison with *C. elegans* and for integrative studies in evolutionary biology (Sommer *et al.* 1996, 2015). Various genomic tools are available, such as annotated genome, genetic transformation, and reverse genetics through CRISPR/Cas9 engineering (Dieterich *et al.* 2008; Schlager *et al.* 2009; Witte *et al.* 2015). In its natural environment, *P. pacificus* often occurs in a necromenic association with scarab beetles, that is, arrested, long-lived dauer larvae stay on a beetle and, after the beetle’s death, the worms exit the dauer stage and flourish on the carcass (Herrmann *et al.* 2007; Meyer *et al.* 2017).

Predatory feeding in *P. pacificus* is dependent on the presence of teeth-like denticles, which are capable of rupturing the cuticle of the prey. Interestingly, *P. pacificus* displays a mouth-form dimorphism, which is influenced by environmental factors (Bento *et al.* 2010; Ragsdale *et al.* 2013). The eurystomatous (Eu) mouth form is characterized by (i) a wide mouth opening; (ii) a large, claw-like dorsal tooth; and (iii) a hook-shaped subventral tooth, which together enable predatory feeding (Figure 1, A and B). In contrast, the stenostomatous (St) form has a narrow mouth opening and a single flint-shaped dorsal tooth, which only allows bacterial feeding and scavenging, but not predation (Figure 1, C and D). In this study, we focus on the predatory feeding behavior of the Eu form to gain insight into the neural regulation of predation.

**Figure 1:**
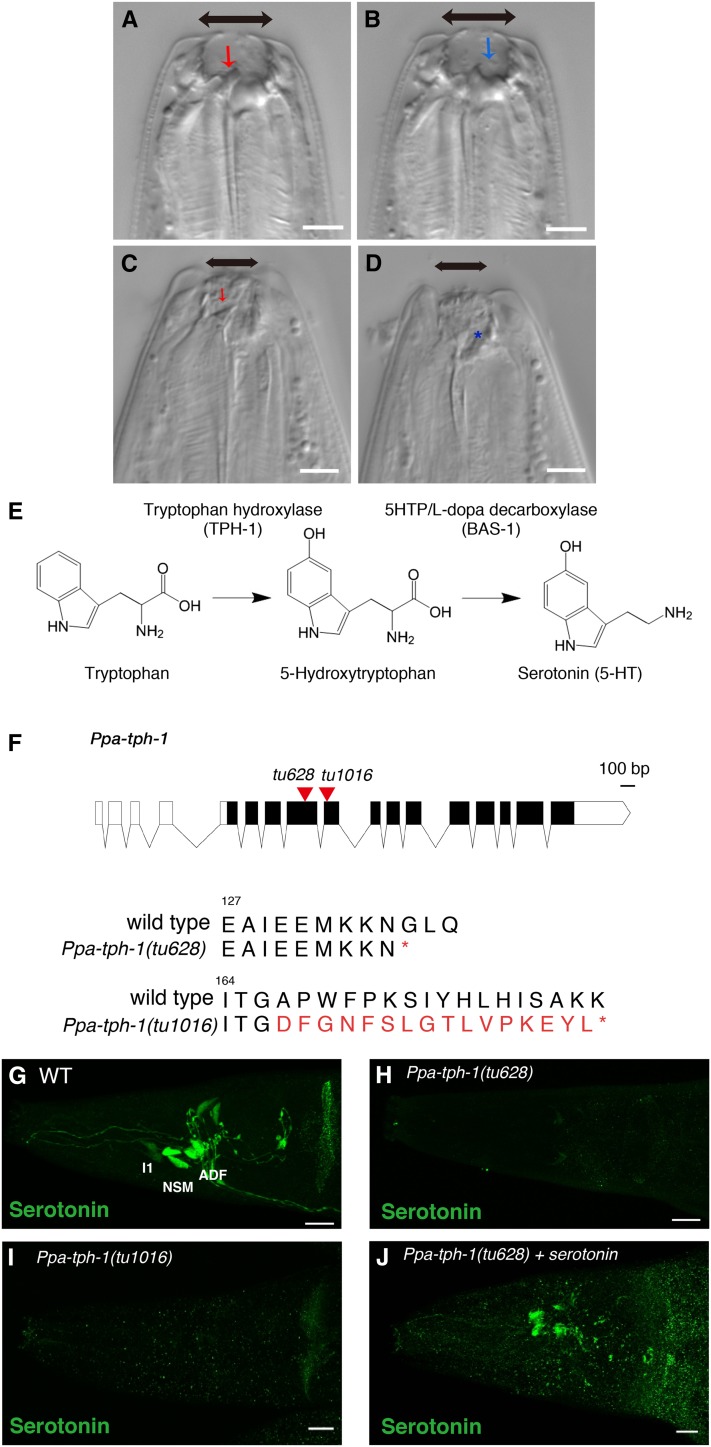
Mouth-form plasticity and generation of *Ppa-tph-1* mutants by CRISPR/Cas9. (A–D) Nomarski images of the mouth dimorphism in *P. pacificus*. Eurystomatous worms have a large mouth opening, a large dorsal tooth (A, red arrow), and a subventral tooth (B, blue arrow). Stenostomatous worms have a narrow mouth opening and a single dorsal tooth (C, red arrow), but no subventral tooth (D, asterisk). Bars are 5 µm. (E) Scheme of serotonin synthesis. Serotonin is synthesized from tryptophan catalyzed by Tryptophan hydroxylase (TPH-1) and 5HTP/L-dopa decarboxylase (BAS-1). (F) Gene structure of *Ppa-tph-1*. Bar is 100 bp. Black rectangles and red triangles show exon–intron structure and the location of CRISPR-induced mutations, respectively. Molecular lesions of both *Ppa-tph-1* mutants result in premature stop codons (asterisks represent stop). (G–J) Projection images of the pharynx of WT (G), *Ppa-tph-1(tu628)* (H), *Ppa-tph-1(tu1016)* (I), and *Ppa-tph-1(tu628)* treated with serotonin (J) stained by anti-serotonin antibody (green). Bars are 10 µm. Serotonin is expressed in NSM, ADF, and I1 neurons in WT but not in the *Ppa-tph-1* mutants. CRISPR, clustered randomly interspersed short palindromic repeats; WT, wild-type.

Previous work identified a feeding mode switch between bacterial feeding and predation in *P. pacificus*. During predation, the rate of pharyngeal muscle contractions termed pumping was reduced by half when compared with bacterial feeding, and additionally tooth movement was stimulated (Wilecki *et al.* 2015). Predatory-like feeding rhythms were also triggered by exogenous serotonin treatment but not by other monoamine neurotransmitters (Wilecki *et al.* 2015). However, the cellular and mechanistic function of serotonin during feeding behaviors in *P. pacificus* remains unknown. Among many animal taxa, pharmacological and genetic studies have shown that serotonin regulates feeding behavior (Gillette 2006). In *C. elegans*, serotonin is synthesized from tryptophan by two conserved enzymes called tryptophan hydroxylase (TPH-1) and 5HTP/L-dopa decarboxylase (BAS-1) (Figure 1E). *C. elegans tph-1* mutants exhibited a reduced pumping rate during bacterial feeding (Sze *et al.* 2000), and serotonin activates pharyngeal pumping and isthmus peristalsis mimicking the effect of bacterial food (Horvitz *et al.* 1982; Avery and Horvitz 1990; Song and Avery 2012).

Here, we investigated the role of serotonin in predatory feeding in *P. pacificus*. We identified the *P. pacificus* tryptophan hydroxylase (*Ppa-tph-1*) and 5HTP/L-dopa decarboxylase (*Ppa-bas-1*), and generated null mutants by utilizing the CRISPR/Cas9 system. *Ppa-tph-1* and *Ppa-bas-1* mutants exhibited a strong reduction in predation efficiency in comparison to wild-type animals. Using high-speed video microscopy, we found that those mutants disrupt the temporal coordination of pharyngeal pumping and tooth movement. Moreover, genetic ablation of the two serotonergic neurons NSM and ADF mimic the phenotype of *Ppa-tph-1* mutants. Our results provide a novel function of serotonin for efficient killing during predation in *P. pacificus* by regulating the coordination of pharyngeal pumping and tooth movement.

## Materials and Methods

### Experimental model

All strains of *P. pacificus* were maintained at 20° on NGM agar plates with *Escherichia coli*. OP50. All experiments were performed using young adult hermaphrodites by picking J4 larvae on the day before the assay. The following mutants and transgenic lines were used: *C. elegans* (N2) and *P. pacificus* (PS312). *Ppa-tph-1(tu628)* (RS2946), *Ppa-tph-1(tu1016)* (RS3074), *Ppa-bas-1(tu629)* (RS2955), *Ppa-bas-1(tu630)* (RS2956), Ex[*Ppa-tph-1p*::*RFP*, *Ppa-egl-20p*::*RFP*] *(tuEx257)* (RS3066), Ex[*Ppa-tph-1p*::*caspase-3(p12)*::*nz*, *Ppa-tph-1p*::*cz*::*caspase-3(p17)*, *Ppa-egl-20p*::*RFP*] *(tuEx259)* (RS3068), and Ex[*Ppa-tph-1p*::*caspase-3(p12)*::*nz*, *Ppa-tph-1p*::*RFP*, *Ppa-egl-20p*::*RFP*] *(tuEx256)* (RS3065).

### Molecular cloning of Ppa-tph-1 and Ppa-bas-1

To identify *Ppa-tph-1* and *Ppa-bas-1*, we utilized phylogenetic analysis between *C. elegans* and *P. pacificus* as previously described (Baskaran *et al.* 2015). Total RNA of the wild-type strain (PS312) was purified using ZR RNA MicroPrep (Zymo Research, R1060). cDNA was synthesized by reverse transcription and used for 5′- and 3′-RACE experiments using the SMARTer RACE 5′/3′ Kit (Clontech, 634858). PCR products were sequenced using Sanger sequencing.

### Generation of Ppa-tph-1 and Ppa-bas-1 mutants by CRISPR/Cas9 system

CRISPR mutants were generated as previously described for *P. pacificus* (Witte *et al.* 2015). To generate *Ppa-tph-1(tu628)*, Cas9 protein (ToolGen Inc.) and sgRNAs (ToolGen Inc.) against *Ppa-tph-1* and *Ppa-dpy-1* were co-injected into PS312 young adults and screened for the dumpy co-CRISPR marker. Dumpy animals were isolated and the F2 were analyzed further for mutations in *Ppa-tph-1*. To obtain an additional *Ppa-tph-1* mutant allele (*tu1016*), we synthesized a new sgRNA using the EnGen sgRNA Synthesis Kit, *S. pyogenes* Cas9 (NEB, #E3322S), and injected PS312 young adult animals. Mutant candidates were obtained by high-resolution melting curve analysis (LightCycler 480 High Resolution Melting Master, Roche, 04909631001) of F1 progeny of injected animals, confirmed by Sanger sequencing. To generate *Ppa-bas-1* mutants, Cas9 protein (ToolGen Inc.) and sgRNA (ToolGen Inc.) against *Ppa-bas-1* were injected into PS312 young adults following the same protocol as for *Ppa-tph-1*.

To generate gRNAs, the following gene-specific sequences were used;

*Ppa-dpy-1*: 5ʹ-TCTCGTGAACGCCAACCGCGTGG-ʹ3.
*Ppa-tph-1(tu628)*: 5ʹ-AGATTCCAAGCAATCGTCATCGG-3ʹ.
*Ppa-tph-1(tu1016)*: 5ʹ-ATGCAATAACGGGAGCTCCTTGG-3ʹ.
*Ppa-bas-1(tu629), Ppa-bas-1(tu630)*: 5ʹ-CTCCTCAGTACCCAGAGGAATGG-3ʹ.

All mutants were backcrossed with PS312 three times before performing further analysis.

### Generation of transgenic lines

A 4.7 kb upstream region of *Ppa-tph-1* was amplified by PCR and fused with *TurboRFP* and *Ppa-rpl-23* 3′-UTR to generate *Ppa-tph-1p*::*RFP*. *Caspase-3 (p12)*::*nz* and *cz*::*caspase-3 (p17)* were amplified from *Pmec-18 caspase-3 (p12)*::*nz [TU#813]* (Addgene #16082) and *Pmec-18 cz*::*caspase-3 (p17) [TU#814]* (Addgene #16083), respectively (Chelur and Chalfie 2007). The *Ppa-tph-1* promoter, each reconstituted caspase subunit, and the *Ppa-rpl-23* 3′-UTR were fused to construct *Ppa-tph-1p*::*caspase-3(p12)*::*nz* and *Ppa-tph-1p*::*cz*::*caspase-3(p17)*, respectively. These transgenes (10 ng/µl), *Ppa-egl-20p*::*TurboRFP* (10 ng/µl) as co-injection maker, and PS312 genomic DNA (60 ng/µl) were digested by the appropriate restriction enzymes and were injected into young adults of PS312 as previously described by Schlager *et al.* (2009).

### Immunostaining

Immunostaining was performed as previously described by Desai *et al.* (1988). Briefly, several plates with adult worms were washed in M9 buffer three times and fixed with 4% paraformaldehyde/PBS at 4° overnight. Samples were washed with 0.5% Triton X-100/PBS (PBST) three times and treated with 5% β-mercaptoethanol/1% TX-100/0.1 M Tris (pH 7.5) overnight at 37°. After being washed in 1% TX-100/0.1 M Tris (pH 7.5) three times and in collagenase buffer [1 mM CaCl_2_/1% TX-100/0.1 M Tris (pH 7.5)], samples were incubated for 1–3 hr at 37° in 2000 Units/ml Collagenase type IV (SIGMA, C5138) in collagenase buffer. The samples were rinsed with PBST three times and blocked at room temperature for 1 hr in 1% BSA/PBST. They were incubated at 4° overnight in the first antibody solution and rinsed with PBST three times. After a second blocking step for 1 hr in 1% BSA/PBST, samples were incubated for 2–4 hr at 37° with the secondary antibody solution. Samples were washed with PBST three times and mounted on agarose pads with Vectashield (Vector Laboratories, Inc., H-1000) for imaging. Images were obtained using a TCS SP8 confocal microscope (Leica) and were processed using ImageJ. The following antibodies were used in this study; anti-serotonin (1:100, rabbit, SIGMA, S5545), anti-RFP (1:500, mouse, Cell Biolabs, AKR-021), goat anti-rabbit IgG secondary antibody Alexa Fluor 488 (1:100, Thermo Fisher, A-11008), and goat anti-mouse IgG Cy3 (1:100, Jackson ImmunoResearch Laboratories Inc. 115-165-146).

### Egg-laying assay

Worms were synchronized by bleaching and single J4 larvae were picked to each plate. Worms were transferred to new plates every 12 hr and the number of eggs was counted. To count the number of eggs laid in short time intervals, 1-d-old adults were transferred to 96-well plates with 50 µl of M9 buffer or 10 mM serotonin solution. After 2 hr incubation at 20°, the number of eggs was counted. The number of eggs in the uterus was counted by observing 3-d-old adults with an Axioplan 2 microscope (Zeiss).

### Corpse assay

The “corpse assay” was performed as previously described (Wilecki *et al.* 2015; Lightfoot *et al.* 2016). To collect young larvae as prey, freshly starved plates of *C. elegans* (N2) were washed with M9 buffer and larvae were filtered twice with 20 µm Nylon Net Filters (Millipore, NY2004700). Young larvae were centrifuged and 3 µl of worm pellet were dropped on an unseeded 60 mm NGM assay plate. Five predators were added to the assay plates. After 2 hr, the number of corpses was counted. Five plates of each strain were tested per experiment and the experiments were repeated three times. The data are the sum of all the experiments.

### Biting assay

The “biting assay” was performed as previously described by Wilecki *et al.* (2015) and Lightfoot *et al.* (2016). In short, young larvae were collected as mentioned in the previous section. Predators were transferred to the assay plate with young larvae of *C. elegans* and they were allowed to recover for 15 min. After recovery, the predator was observed using a light stereomicroscope for 10 min and the numbers of biting, killing, and feeding events were counted. Per experiment, 4–7 animals of each strain were tested, and the experiments were repeated two or three times. The data are the sum of all the experiments.

### Pumping and tooth movement

Pumping and tooth movement were observed as previously described by Wilecki *et al.* (2015) and Lightfoot *et al.* (2016). Worms were transferred to 35 mm NGM assay plates with *E. coli* OP50 or plates full with young larvae of *C. elegans* (N2). After 15 min recovery time, pharyngeal pumping and tooth movement were video-recorded using a high-speed camera (Axio Imager A1, Zeiss) with 50 Hz for 15 sec. For treatment with serotonin or dopamine, worms were incubated in 5 mM solutions of the neurotransmitters for 4 hr and pharyngeal pumping and tooth movement were observed within 20 min after transferring worms onto assay plates. The timing of tooth closure and muscle relaxation were observed and plotted as tooth movement and pumping, respectively.

### Statistical analysis

**Figure 2:**
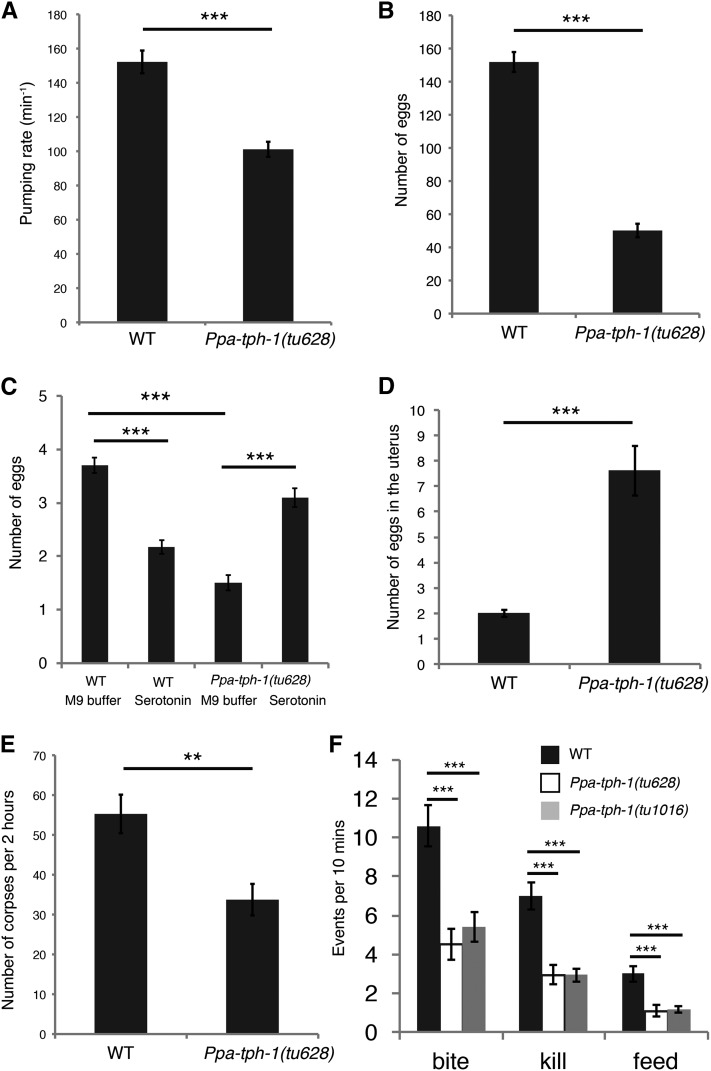
Mutations in *Ppa-tph-1* reduce predatory feeding behavior. (A) Pharyngeal pumping rates of wild-type (WT) and *Ppa-tph-1(tu628)* mutants on *E. coli* OP50. *n* = 20, Error bars are SEM. Two-sided Welch’s *t*-test. *** *p* < 2.9*10^−7^. (B) Number of eggs laid within 1 wk. WT, *n* = 48; *Ppa-tph-1(tu628)*, *n* = 46. Error bars are SEM. Two-sided Welch’s *t*-test. *** *p* < 2.2*10^−16^. (C) Number of eggs laid in short time intervals. Worms were transferred to M9 buffer or 10 mM serotonin solution and incubated for 2 hr. *n* = 102 for each condition and genotype. Error bars are SEM. Two-way ANOVA with two-sided Tukey’s multiple comparison tests. *** *p* < 1.0*10^−7^. (D) Number of eggs in the uterus of 3-d-old adult hermaphrodites. WT, *n* = 50; *Ppa-tph-1(tu628)*, *n* = 44. Error bars are SEM. Two-sided Welch’s *t*-test. *** *p* < 1.1*10^−6^. (E) Number of corpses of *C. elegans* larvae after 2 hr incubations with five *P. pacificus* predators. *n* = 15. *n* represents the number of plates. Error bars are SEM. Two-sided Welch’s *t*-test. ** *p* < 0.01. (F) Number of predation events in 10 min. *n* = 20. Error bars are SEM. One-way ANOVA with two-sided Tukey’s multiple comparison tests. *** *p* < 1.0*10^−3^.

Data were processed and plotted using Excel and R 3. 2. 1 and statistical analysis was performed using R 3. 2. 1. All data are represented as mean ± SEM unless otherwise noted. Statistical details of experiments, including statistical tests, statistical significance, and the exact *n* numbers, can be found in the figure legends. “*n*” is denoted as the number of animals used except for the corpse assay (Figure 2E). When *p* values were < 0.05, the results were considered to be statistically significant. No statistical methods were used to predetermine sample size. The experiments were not randomized, and the investigators were not blinded to allocation during experiments and outcome assessment.

### Data availability

All materials and data used in this study, including mutant, transgenic lines, and plasmids, are available upon request from the corresponding authors.

## Results

### CRISPR/Cas9-induced Ppa-tph-1 mutants show conserved functions in pharyngeal pumping and egg laying

To study the involvement of serotonin in predatory feeding behavior, we generated mutants in the tryptophan hydroxylase (*Ppa-tph-1*) gene of *P. pacificus* using the CRISPR/Cas9 system because TPH-1 is an essential enzyme for serotonin biosynthesis (Figure 1, E and F). We obtained two independent alleles of *Ppa-tph-1*: *tu628* with a 10 bp insertion, and *tu1016* with a 2 bp deletion and a 24-bp insertion that resulted in a premature stop codon (Figure 1F). To confirm that the mutant alleles are indeed nonfunctional, we performed immunostaining experiments with an anti-serotonin antibody. In wild-type animals, serotonin is expressed in three pairs of neurons (NSM, ADF, and I1) in the head region and in the hermaphrodite-specific neurons (HSN) in the vulva region (Wilecki *et al.* 2015; Figure 1G). Serotonin expression was completely absent in *Ppa-tph-1(tu628)* and *Ppa-tph-1(tu1016)* mutants (Figure 1, H and I). Thus, both *Ppa-tph-1* alleles are unable to produce serotonin and are likely strong reduction-of-function or null alleles.

In *C. elegans*, serotonin is involved in feeding behavior and *Cel-tph-1* mutants show a reduction in the rate of pharyngeal pumping on bacterial food (Sze *et al.* 2000). We examined the pumping rate during bacterial feeding in *P. pacificus* to identify if this function of serotonin is conserved between both species. We found a significantly lower pumping rate of *Ppa-tph-1* mutants in comparison to wild-type (*p* < 2.9*10^−7^), indicating that serotonin controls the pumping rate during bacterial feeding in *P. pacificus* (Figure 2A). As *C. elegans* serotonin expression in the HSN neurons regulates egg-laying behavior (Sze *et al.* 2000), we also investigated its potential role in *P. pacificus*. The lifetime fecundity of the *P. pacificus* wild-type reference strain (PS312) is ∼150 progeny, whereas *Ppa-tph-1* mutants showed a significant reduction with an average of 50 progeny (Figure 2B). We also counted egg laying after a 2 hr treatment with serotonin or M9 buffer control treatments. Although the serotonin treatment suppressed egg laying in wild-type animals, confirming previous results (Gutierrez and Sommer 2007), it partially rescued the egg-laying defect in *Ppa-tph-1* mutant animals (Figure 2C, *p* < 1.0*10^−7^). The number of eggs *in utero* also increased in *Ppa-tph-1(tu628)*, resulting in an egg-laying-defective (Egl) phenotype in 80% of mutant adults (Figure 2D). In contrast, wild-type *P. pacificus* eggs are usually released in the four- or eight-cell stages and an Egl phenotype is observed extremely rarely. Together, these data suggest that the function of serotonin during pharyngeal pumping is conserved between *P. pacificus* and *C. elegans*, and that a proper amount of serotonin is important for egg laying in *P. pacificus*.

### Ppa-tph-1 is required for efficient killing during predation

Next, we set out to test the involvement of serotonin in predatory feeding. We performed two killing assays, corpse assays and biting assays, that were established previously (Wilecki *et al.* 2015; Lightfoot *et al.* 2016). In short, in corpse assays, five *P. pacificus* adults are incubated as predators with thousands of *C. elegans* L2 larvae as prey on unseeded NGM plates and the number of corpses are counted after a 2 hr time period. Comparing the *Ppa-tph-1* mutants with wild-type animals, we found a strong reduction in the number of corpses; however, corpses were still detectable and not completely eliminated (Figure 2E). Specifically, five wild-type predators kill between 50 and 60 *C. elegans* larval prey, whereas *Ppa-tph-1* mutants kill ∼30 animals. In order to examine the predatory behavior in greater detail, we conducted bite assays. For this, one predator is observed over a 10 min time interval and the number of biting, killing, and feeding events on prey larvae are quantified. We observed that *Ppa-tph-1* mutants bit and killed less prey than wild-type animals (Figure 2F). In addition, the number of feeding events was also decreased in *Ppa-tph-1* mutants. Together, these results suggest that serotonin is required for efficient killing during predation and that the absence of serotonin reduces, but does not completely eliminate, predatory killing and feeding behavior.

### Serotonin is necessary for the coordination of pharyngeal pumping and tooth movement

**Figure 3:**
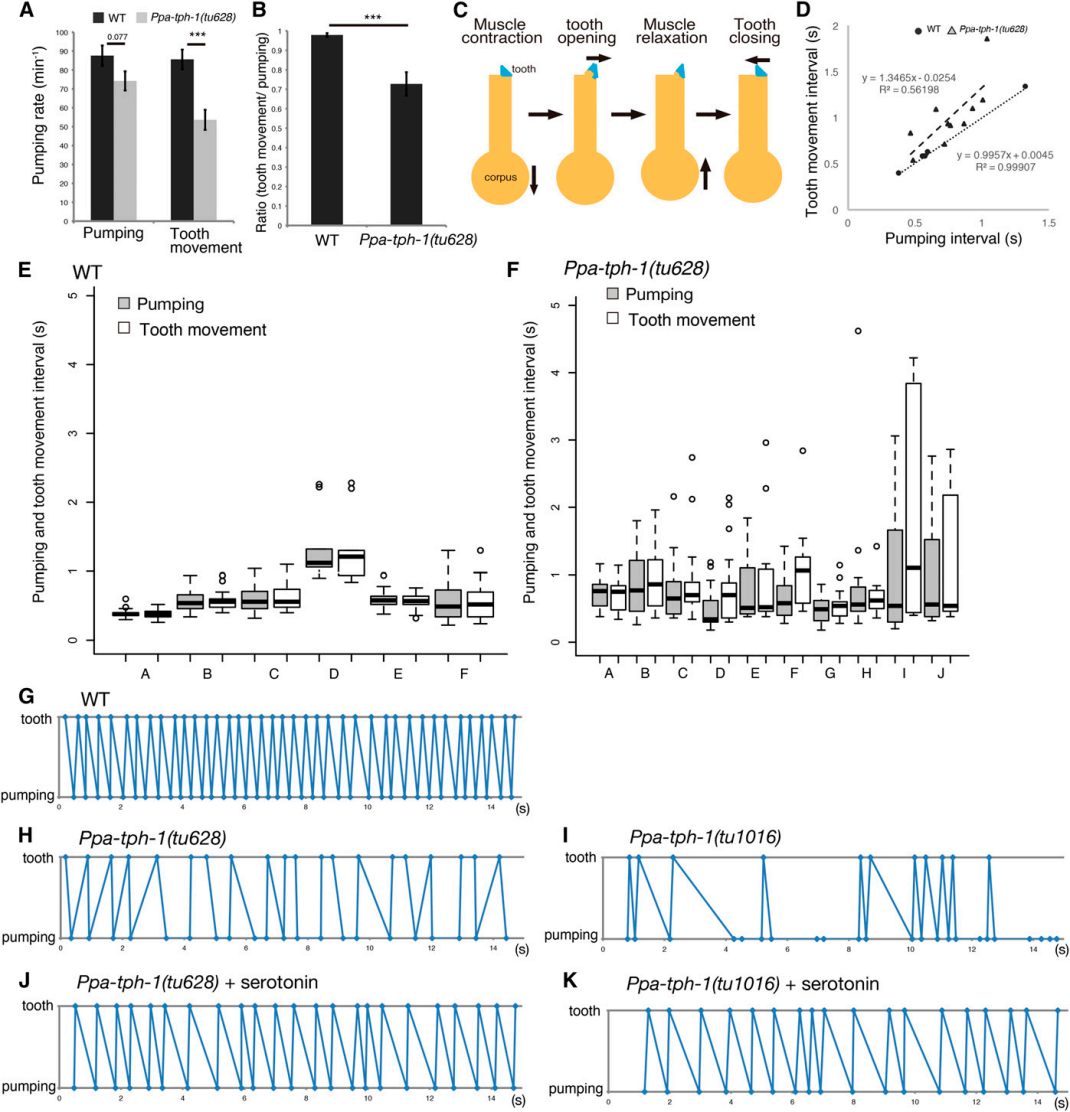
Serotonin is required for the correct timing of pumping and tooth movement. (A) Pumping rate and tooth movement during the predatory feeding behavior. *n* = 20, Error bars are SEM. Two-sided Welch’s *t*-test. *** *p* < 1.1*10^−4^. (B) Ratio of pumping and tooth movement. *n* = 20, Error bars are SEM. Two-sided Welch’s *t*-test. *** *p* < 5.1*10^−4^. (C) Scheme of pharyngeal pumping and tooth movement. The yellow outline represents the anterior part of the pharynx, the blue triangles indicate the tooth. (D) Relationship between pumping intervals and tooth movement intervals using Pearson’s correlation coefficient test. Circle and triangle represent the individual average of the intervals in wild-type (WT) and *Ppa-tph-1(tu628)*, respectively. (E and F) Boxplots show the individual variables of the pumping interval (gray) and the tooth movement interval (white) in WT (E) and *Ppa-tph-1(tu628)* mutant animals (F). Box and dark line within the box represent upper and lower quartiles and median, respectively. The whiskers (dashed lines) show the 1.5× quantile range and circles are outlier of this range. Items on the *x*-axis correspond to the individual results of Figure S1 and Figure S2 in File S1. (G–K) Representative time plots of tooth closure (plotted as tooth) and muscle relaxation of corpus (plotted as pumping) in 15 sec during predation. (G) WT, (H) *Ppa-tph-1(tu628)*, (I) *Ppa-tph-1(tu1016)*, (J) *Ppa-tph-1(tu628)* treated with serotonin, and (K) *Ppa-tph-1(tu1016)* treated with serotonin. All results of the other individuals are shown in Figure S1, Figure S2, Figure S3, Figure S4, and Figure S5 in File S1.

As serotonin appeared to be involved in the efficiency of predatory feeding, we investigated its mechanistic function in more detail. For that, we quantified the feeding rhythm during predation using a high-speed camera system. We found that in *P. pacificus*, pharyngeal pumping of wild-type animals decreased during predation maintaining an ∼1:1 ratio of pumping and tooth movement (Figure 3, A and B), confirming previous observations (Wilecki *et al.* 2015). In *Ppa-tph-1* mutants, pharyngeal pumping was often similar, but tooth movement was significantly lower than in wild-type animals (Figure 3A, *p* < 1.1*10^−4^). Similarly, the ratio of pharyngeal pumping to tooth movement was also decreased in *Ppa-tph-1* mutants (Figure 3B, *p* < 5.1*10^−4^).

Strikingly, we observed a novel role for serotonin in controlling the temporal coordination of pharyngeal pumping and tooth movement when analyzing the predation rhythms in more detail (Figure 3C). During predation, the dorsal tooth and the pharyngeal muscles move rhythmically. First, the muscles contract while the tooth opens, then, the muscles relax and finally the tooth closes. We plotted the timing of tooth closure and muscle relaxation to characterize the coordination between tooth movement and pharyngeal pumping, respectively (Figure 3, G–K and Supplemental Material, Figure S1, Figure S2, Figure S3, Figure S4, and Figure S5 in File S1), and quantified the interval between two subsequent pumping events (pumping interval) and the interval between two subsequent tooth movements (tooth movement interval) (Figure 3, D–F). In wild-type animals, the timing of pumping and tooth movement was highly coordinated with a strict 1:1 ratio (Figure 3, D, E, and G and Figure S1 in File S1) and the interval was almost constant within individuals (Figure 3E). In contrast, timing of pumping and tooth movement in both *Ppa-tph-1* mutant alleles were completely uncoordinated with several defects (Figure 3, D, F, H, and I, Figure S2, and Figure S3 in File S1). First, tooth closure was seen before muscle relaxation, a pattern never seen in wild-type animals (Figure 3H and Figure S1 in File S1). Second, two pharyngeal pumping events could occur without intermediate tooth movements (Figure 3, D, H, and I, Figure S2, and Figure S3 in File S1). Finally, the intervals of pumping and tooth movement were not coordinated with a 1:1 ratio (Figure 3D) and the variance of pumping and tooth movement intervals within an individual were larger than that of wild-type (Figure 3F), resulting in the loss of temporal coordination of predation.

Next, we examined whether exogenous serotonin would restore the defects observed in *Ppa-tph-1* mutants. For this, *P. pacificus* predators were incubated in 5 mM serotonin solution for 4 hr and moved to assay plates with *C. elegans* larvae as prey. Such serotonin treatment was sufficient for the reuptake of serotonin into neurons, as confirmed by immunostaining with the anti-serotonin antibody (Figure 1J). Indeed, *Ppa-tph-1* mutants treated with serotonin restored pharyngeal pumping and tooth movement to nearly wild-type levels (Figure 3, J and K, Figure S4, and Figure S5 in File S1). Taken together, these results support the role of serotonin as a major regulator of the temporal coordination of pharyngeal pumping and tooth movement.

### Ppa-bas-1 mutants mimic the phenotypes of Ppa-tph-1 mutants

**Figure 4:**
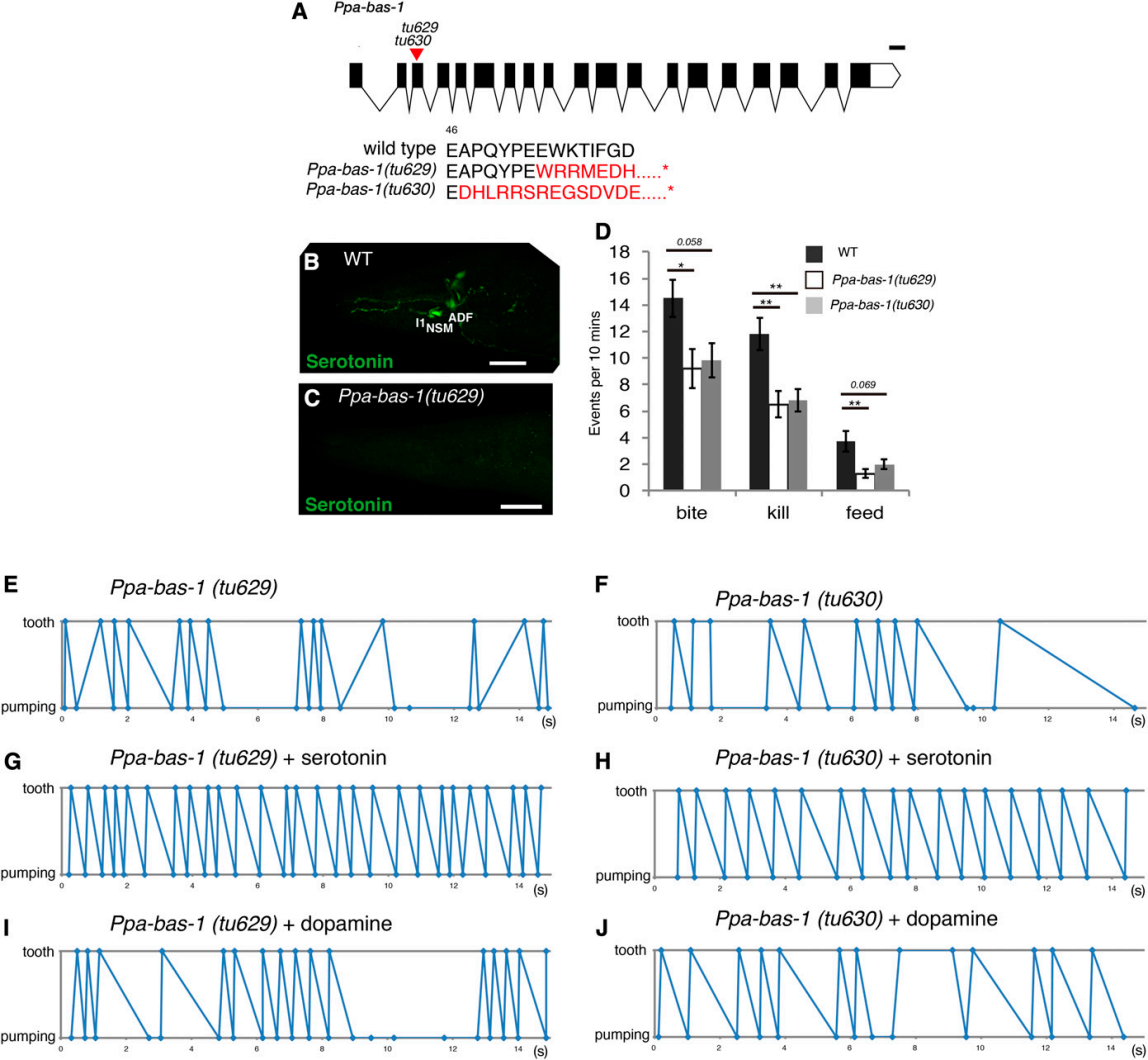
*Ppa-bas-1* mutants showed the uncoordinated timing of pumping and tooth movement. (A) Gene structure of *Ppa-bas-1*. Bar is 100 bp. Black rectangles and a red triangle show exon–intron structure and the location of the CRISPR-induced mutations, respectively. Molecular lesions of both *Ppa-bas-1* mutants result in premature stop codons (asterisks represent stop). (B and C) Projection images of the pharynx of WT (B) and *Ppa-bas-1(tu629)* mutants (C) stained by anti-serotonin antibody (green). Bars are 20 µm. (D) Number of predatory events in 10 min. *n* = 10. Error bars are SEM. One-way ANOVA with two-sided Tukey’s multiple comparison tests. * *p* < 0.05 and ** *p* < 0.01. (E–J) Representative time plots of tooth closure (plotted as tooth) and muscle relaxation of corpus (plotted as pumping) in 15 sec during predation. (E); *Ppa-bas-1(tu629)*, (F); *Ppa-bas-1(tu630)*, (G); *Ppa-bas-1(tu629)* treated with serotonin, (H); *Ppa-bas-1(tu630)* treated with serotonin, (I); *Ppa-bas-1(tu629)* treated with dopamine, and (J); *Ppa-bas-1(tu630)* treated with dopamine. All results of other individuals are shown in Figure S6, Figure S7, Figure S8, Figure S9, Figure S10, and Figure S11 in File S1. CRISPR, clustered randomly interspersed short palindromic repeats; WT, wild-type.

To provide additional evidence for the role of serotonin in the regulation of predatory feeding behavior, we generated a mutant in the 5HTP/L-dopa decarboxylase gene *Ppa-bas-1*, which is required for the synthesis of serotonin and dopamine (Figure 1E and Figure 4A). We obtained two mutant alleles, *tu629* with a 1 bp deletion and an 8 bp insertion, and *tu630* with a 26 bp deletion, both of which resulted in a premature stop codon (Figure 4A). Immunostaining using the anti-serotonin antibody revealed that, similar to *Ppa-tph-1*, serotonin was completely absent in *Ppa-bas-1* mutants, indicating that these mutant worms were unable to produce serotonin (Figure 4, B and C). Biting assays performed with *Ppa-bas-1* mutants showed that killing was significantly reduced compared to wild-type (Figure 4D). Most importantly, the coordination of pharyngeal pumping and tooth movement was also disrupted in *Ppa-bas-1* mutants (Figure 4, E and F, Figure S6, and Figure S7 in File S1). Finally, as with *Ppa-tph-1* mutants, the coordination of tooth movement and pumping could be restored by serotonin treatment, but not by treatment with dopamine (Figure 4, G–J, Figure S8, Figure S9, Figure S10, and Figure S11 in File S1). These results further support the conclusion that serotonin controls temporal coordination of pharyngeal pumping and tooth movement. In addition, these results indicate that dopamine is not involved in regulating predatory behavior.

### Reconstituted caspases are able to induce cell death in P. pacificus

**Figure 5:**
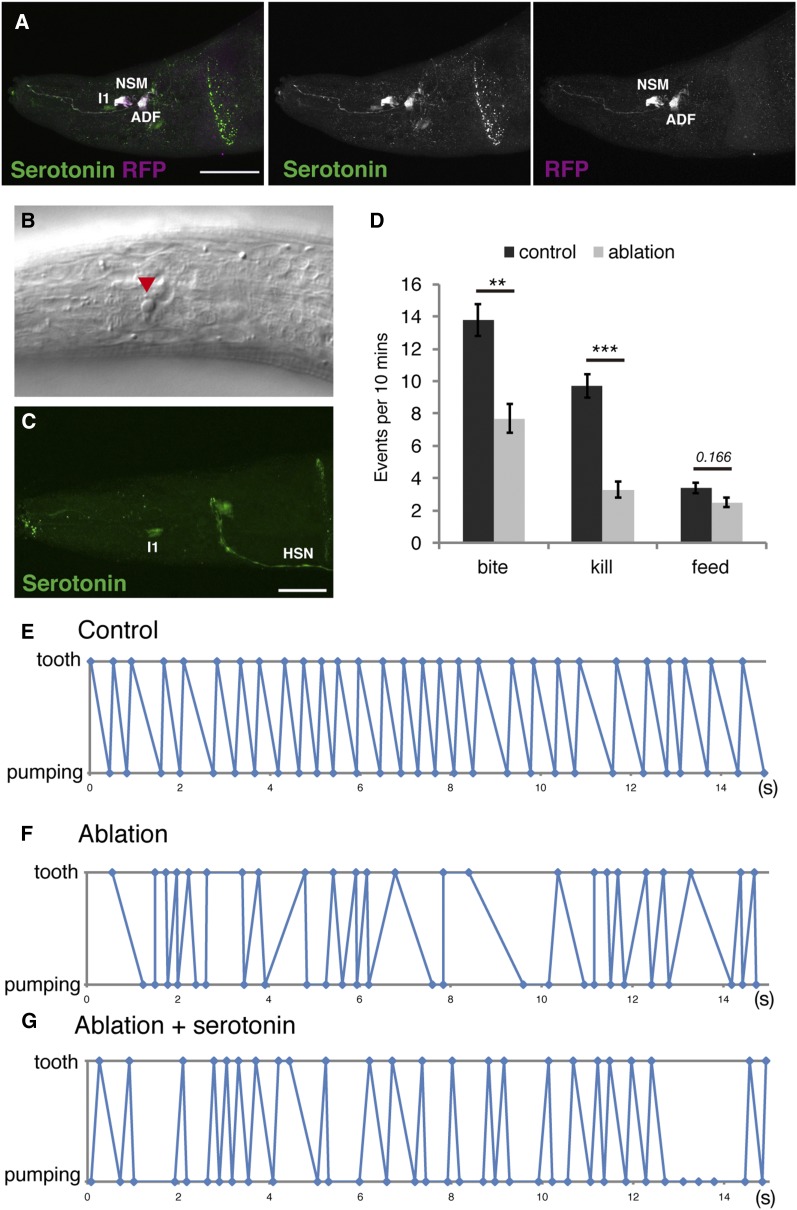
Genetic ablation of NSM and ADF neurons caused the abnormal timing of pumping and tooth movement. (A) Projection images of worms expressing *Ppa-tph-1p*::*RFP*. Green and magenta show serotonin and RFP staining, respectively, with RFP being expressed in NSM and ADF neurons. Bar is 30 µm. (B) Representative image of cell death (red triangle) induced by reconstituted caspases being driven by the *Ppa-tph-1* promoter. (C) Projection image of the pharynx of worms expressing reconstituted caspases driven by the *Ppa-tph-1* promoter with serotonin staining in green. Bar is 20 µm. (D) Number of predatory feeding events in 10 min. Worms expressing a single reconstituted caspase subunit are used as control. *n* = 10, Error bars are SEM. Two-sided Welch’s *t*-test. ** *p* < 0.01 and *** *p* < 9.5*10^−5^. (E–G) Representative time plots of tooth closure (plotted as tooth) and muscle relaxation of corpus (plotted as pumping) in 15 sec during predation. (E) control, (F) NSM, and (G) ADF ablation; NSM- and ADF-ablated worms treated with serotonin. All results of other individuals are shown in Figure S12, Figure S13, and Figure S14 in File S1. HSN, hermaphrodite-specific neurons; RFP, red fluorescent protein.

Next, we wanted to identify which neurons are responsible for the coordination of pharyngeal pumping and tooth movement. To address this question, we established a genetic cell ablation system in *P. pacificus*. We utilized the reconstituted caspases consisting of the small and large subunits of human caspase-3 with zinc-finger domains (Chelur and Chalfie 2007). Cells expressing both subunits undergo cell death, whereas the expression of a single subunit does not induce cell death. To express the reconstituted caspases in serotonin-positive neurons, we generated transcriptional reporter lines of *Ppa-tph-1* using a 4.7 kb upstream region of *Ppa-tph-1*. The *Ppa-tph-1p*::*RFP* reporter construct was expressed in a subset of neurons in the head region (Figure 5A). We performed immunostaining with anti-serotonin and anti-RFP antibodies and found that RFP is expressed in NSM and ADF neurons, but not in I1 and HSN neurons, in both Eu and St mouth-form animals (Figure 5A). Using the same *Ppa-tph-1* promoter, we expressed both subunits of the reconstituted caspase in NSM and ADF neurons to study if serotonin expression in these cells is sufficient for the function of serotonin in predatory feeding. When expressing the reconstituted caspases under the *Ppa-tph-1* promoter, we observed cell debris with a disk-like appearance that is characteristic of apoptotic cells (Figure 5B). When we performed anti-serotonin immunostaining, a serotonin signal was only observed in I1 and HSN, indicating that the NSM and ADF neurons were indeed absent in worms expressing the reconstituted caspases (Figure 5C). These results indicate that the reconstituted caspases can induce cell death in *P. pacificus*, providing a powerful new method for mechanistic studies in *P. pacificus*.

### Genetic ablation of NSM and ADF neurons mimic the coordination defects of the Ppa-tph-1 and Ppa-bas-1 mutants

Using the reconstituted caspase system, we ablated NSM and ADF neurons and performed biting assays to investigate if these neurons are involved in regulating the predatory behavior. If serotonin function in NSM and/or ADF is involved in the regulation of predatory feeding, genetic ablation of both cells should result in uncoordinated tooth movement and pharyngeal pumping similar to *Ppa-tph-1* mutants. In contrast, if serotonin function in I1 is responsible for the regulation of predatory feeding, genetic ablation of NSM and ADF should not result in uncoordinated timing of pharyngeal pumping and tooth movement. We found that the ablation of NSM and ADF neurons decreased the numbers of biting and killing compared with control worms that express only a single subunit and do not show any cell death (Figure 5D). Feeding was not affected by the ablation of NSM and ADF. We also found that the timing and dynamics of the pharyngeal pumping and tooth movement were uncoordinated in ablated worms, similar to the phenotype of *Ppa-tph-1* and *Ppa-bas-1* mutants (Figure 5, E and F, and Figure S12 and Figure S13 in File S1). Interestingly, exogenous treatment with 5 mM serotonin did not rescue the phenotype in genetically-ablated animals (Figure 5G and Figure S14 in File S1). Taken together, these results indicate first that ADF and/or NSM are required for the temporal coordination of pharyngeal pumping and tooth movement during predation, and second that the disrupted connectivity of ADF and/or NSM-containing neuronal circuits control feeding rhythm in *P. pacificus*.

## Discussion

Serotonin is a neurotransmitter regulating feeding behaviors in many animals. In this study, we investigated the functions of serotonin during predatory feeding in *P. pacificus*. Our finding that the major function of serotonin during predation in *P. pacificus* is the coordination of feeding rhythms is based on three independent observations. First, mutants in *Ppa-tph-1* and *Ppa-bas-1* disrupt feeding rhythms resulting in inefficient feeding behavior. Second, exogenous serotonin treatment can largely restore a wild-type feeding rhythm in *Ppa-tph-1* and *Ppa-bas-1* mutants. Third, genetic cell ablation utilizing the reconstituted caspases in *P. pacificus* revealed that the ablation of NSM and ADF neurons also exhibited uncoordinated pumping and tooth movement, suggesting that NSM and/or ADF neurons play a key role in this process. The finding that serotonin treatment did not restore the phenotypes in genetically-ablated animals suggests that serotonin may act on these neurons themselves. Alternatively, serotonin may allow the NSM and/or ADF neurons to send signals to downstream targets via other neurotransmitters or neuropeptides.

A previous study showed that exogenous treatment of serotonin triggers a predatory-like feeding mode, characterized by a slower pumping rate and tooth movement (Wilecki *et al.* 2015). In contrast, the loss of serotonin also decreased pumping rate during bacterial feeding compared to wild-type. This complex regulation of pumping rate may be explained by feeding mode switching between bacterial and predatory feeding behaviors mediated by serotonin. As exogenous serotonin treatment induces the predatory feeding mode without prey items, the sensory information from the nematode prey such as odor, taste, or touch might activate the serotonin-signaling pathway and switch the pharyngeal muscle state from the bacterial feeding mode to the predatory feeding mode. Given that mutations in *Ppa-tph-1* did not abolish predation completely and did not reduce the pumping rate compared to wild-type during predation, it is likely that there is another pathway that mediates switching mode, especially affecting pharyngeal pumping. In *C. elegans*, sensory information such as light inhibits pumping and changes pharyngeal motions in the opposite direction from pumping to spitting (Bhatla *et al.* 2015). In addition, the sensory cues of bacterial food affect the expression level of *tph-1* in ADF neurons and modulate the feeding behavior in *C. elegans* (Song *et al.* 2013). Therefore, we hypothesize further that in *P. pacificus*, sensory information of nematode prey items might activate serotonin and other neural pathways, which eventually induce the switch to the predatory feeding mode.

We showed that serotonin has several functions in *P. pacificus* such as egg laying, stimulating the pumping rate during bacterial feeding, and the coordination of pumping and tooth movement. These independent functions of serotonin might be regulated by several serotonin receptors. In *C. elegans*, there are five serotonin receptors, including four G protein-coupled receptors (SER-1, SER-4, SER-5, and SER-7) and one serotonin-gated chloride channel (MOD-1), that mediate different behaviors (Olde and McCombie 1997; Hamdan *et al.* 1999; Ranganathan *et al.* 2000; Hobson *et al.* 2003; Hapiak *et al.* 2009; Harris *et al.* 2009). Future analysis of the expression pattern and mutants of serotonin receptors in *P. pacificus* might reveal the downstream targets of NSM and ADF neurons, and functions of serotonin during predation. However, such studies are sophisticated as there is no 1:1 orthology between genes encoding receptors in multi-gene families (Markov *et al.* 2015), and therefore such studies will likely require a system-wide analysis of all potential genes of a given gene family.

As discussed above, there are additional neural circuits involved in predatory feeding behavior and switching the two feeding modes in response to sensory cues. In future studies, the CRISPR/Cas9 system can be used to generate mutants in other neurotransmitters, receptors, and neural circuits that further regulate predatory feeding behavior. Additionally, the establishment of a cassette of promoter constructs for driving expression in specific neurons and pharyngeal muscles can be used for genetic cell ablation studies. The establishment of genetic cell ablation as a new *P. pacificus* tool in this study provides a powerful new method for the analysis and identification of neural circuits. In addition, recent analysis with genetic tools and microfluidic devices to modulate and monitor the neural and muscle activities in *C. elegans* made it possible to examine the precise functions of the nervous system in the regulation of feeding (Kerr *et al.* 2000; Lockery *et al.* 2012; Bhatla and Horvitz 2015; Bhatla *et al.* 2015; Trojanowski *et al.* 2016). In the future, such techniques might be applicable to *P. pacificus* and would open new avenues for investigating complex behaviors in *P. pacificus* that are unknown from *C. elegans*.

Finally, it should be noted that future analysis of predatory feeding in *P. pacificus* has to involve additional trajectories. In this study, we have focused on the predatory behavior of the Eu mouth-form animals. As *P. pacificus* has a behavioral dimorphism depending on the alternative mouth forms, there might be neural differences between the two mouth forms that mediate these behavioral differences. For example, there might be differences in the expression pattern of neurotransmitters or receptors, the synaptic connectivity, and/or neural activities, which regulate predatory *vs.* bacterial feeding behaviors. Therefore, comparative studies between the two *P. pacificus* mouth forms, as well as *C. elegans* and other nematodes, can provide new insight into the mechanism driving the evolution of the behavioral novelty.

## Supplementary Material

Supplemental material is available online at www.g3journal.org/lookup/suppl/doi:10.1534/g3.117.300263/-/DC1.
