# Supplementary material for: Serotonin Drives Predatory Feeding Behavior via Synchronous Feeding Rhythms in the Nematode Pristionchus pacificus
Source: G3 (Bethesda). 2017 Sep 12;7(11):3745–55. doi: 10.1534/g3.117.300263 (PMC5677172; doi:10.1534/g3.117.300263)
Supplement: Supplementary file 1 [file 3745FileS1.pdf]

Figure S1

wild type

A

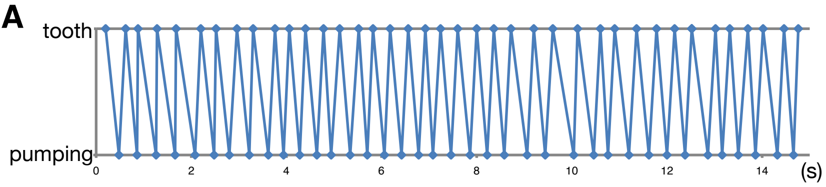

B

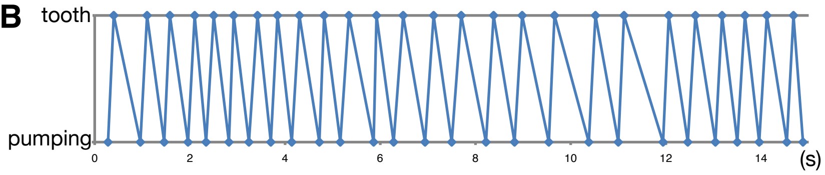

C

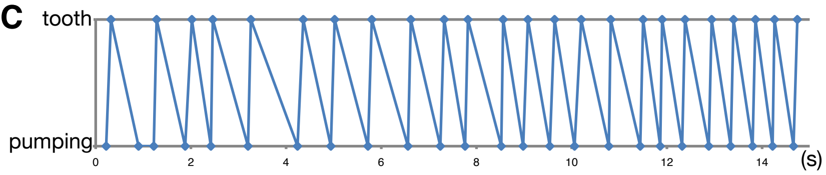

D

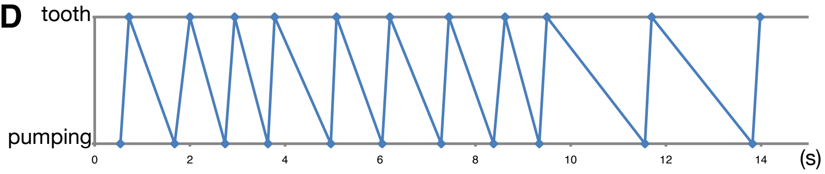

E

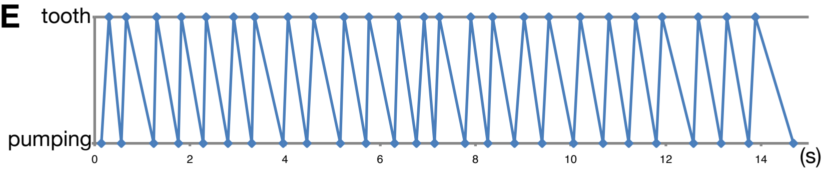

F

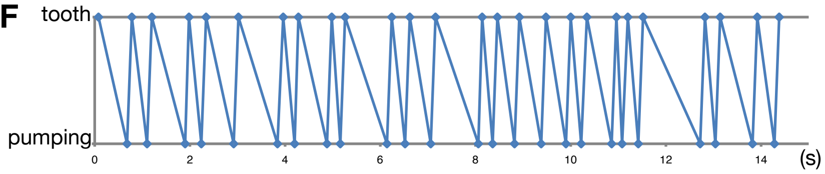

Figure S2

Ppa-tph-1(tu628)

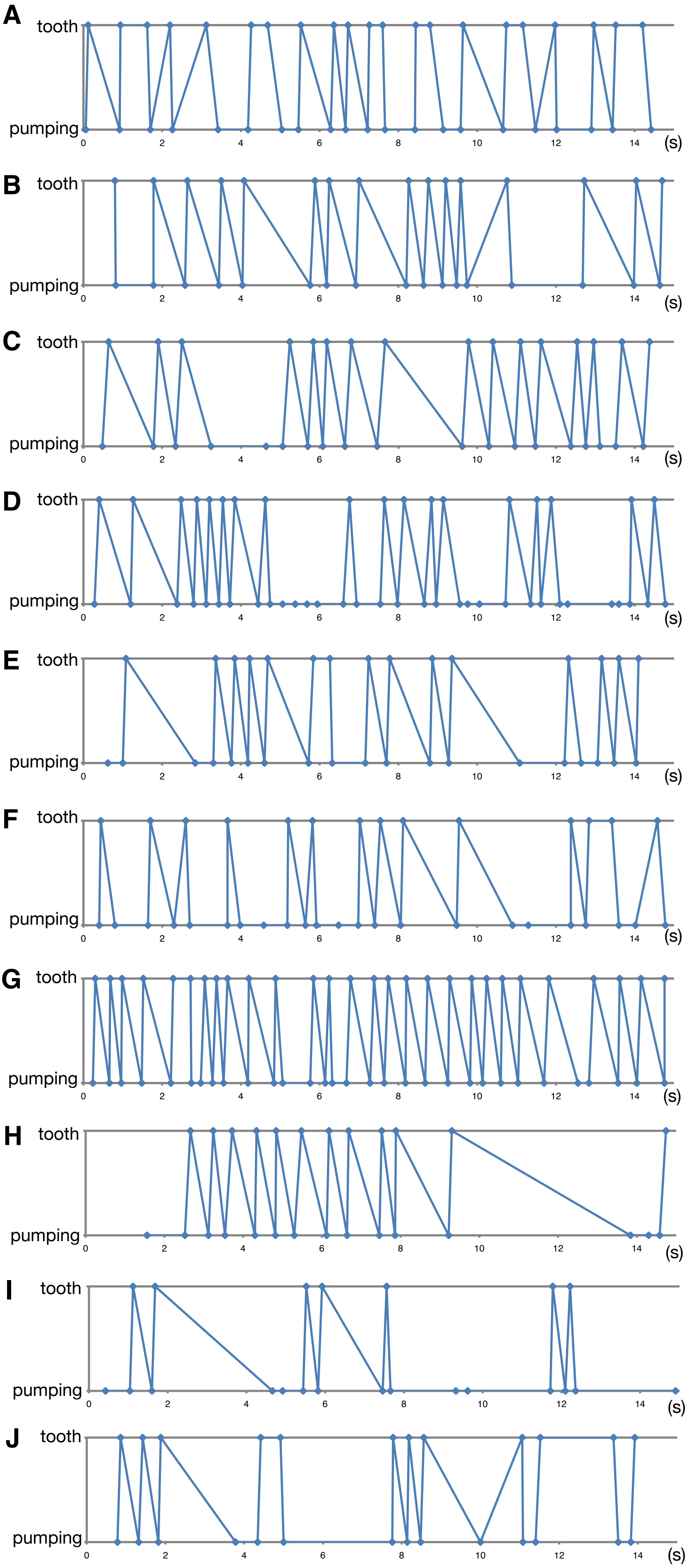

Figure S3

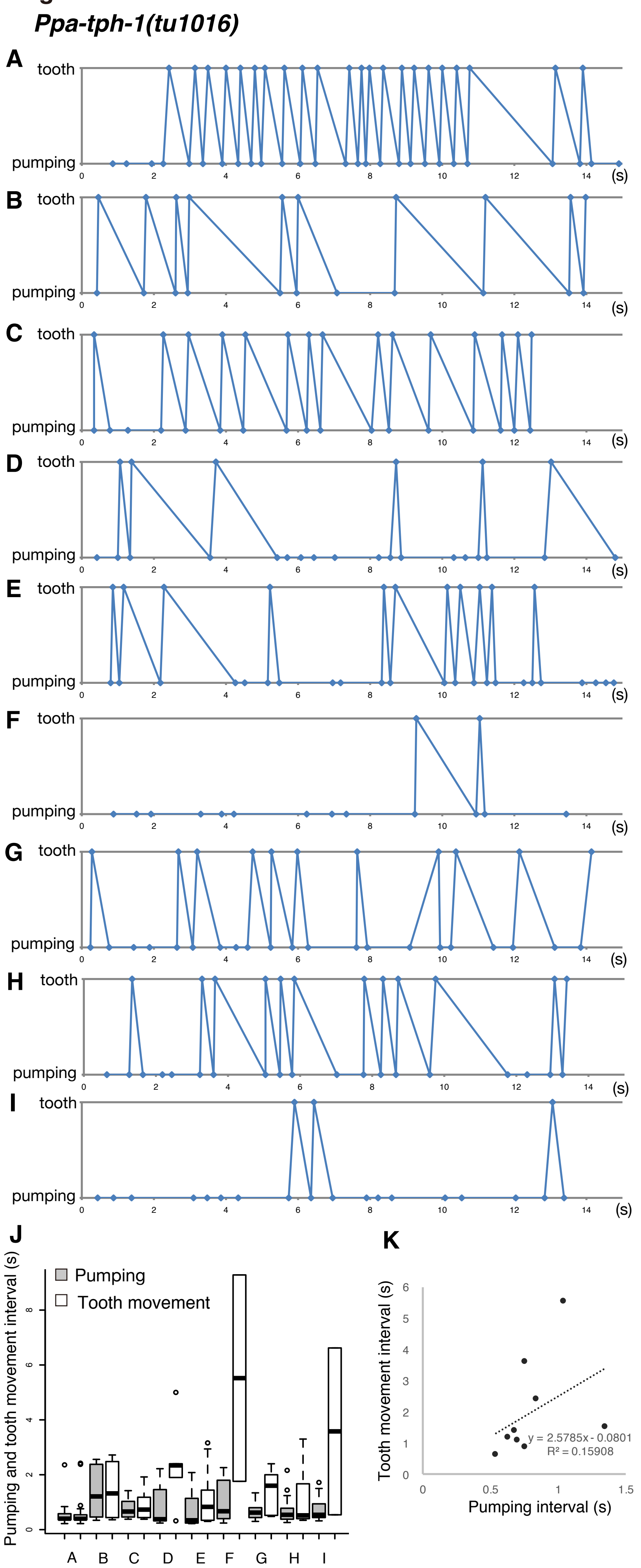

Ppa-tph-1(tu1016)
A
B
C
D
E
F
G
H
I
tooth
pumping
0
2
4
6
8
10
12
14
(s)
J
K
Pumping
Tooth movement
Pumping and tooth movement interval (s)
Tooth movement interval (s)
Pumping interval (s)
y = 2.5785x - 0.0801
R² = 0.15908

Figure S4

Ppa-tph-1(tu628) + serotonin

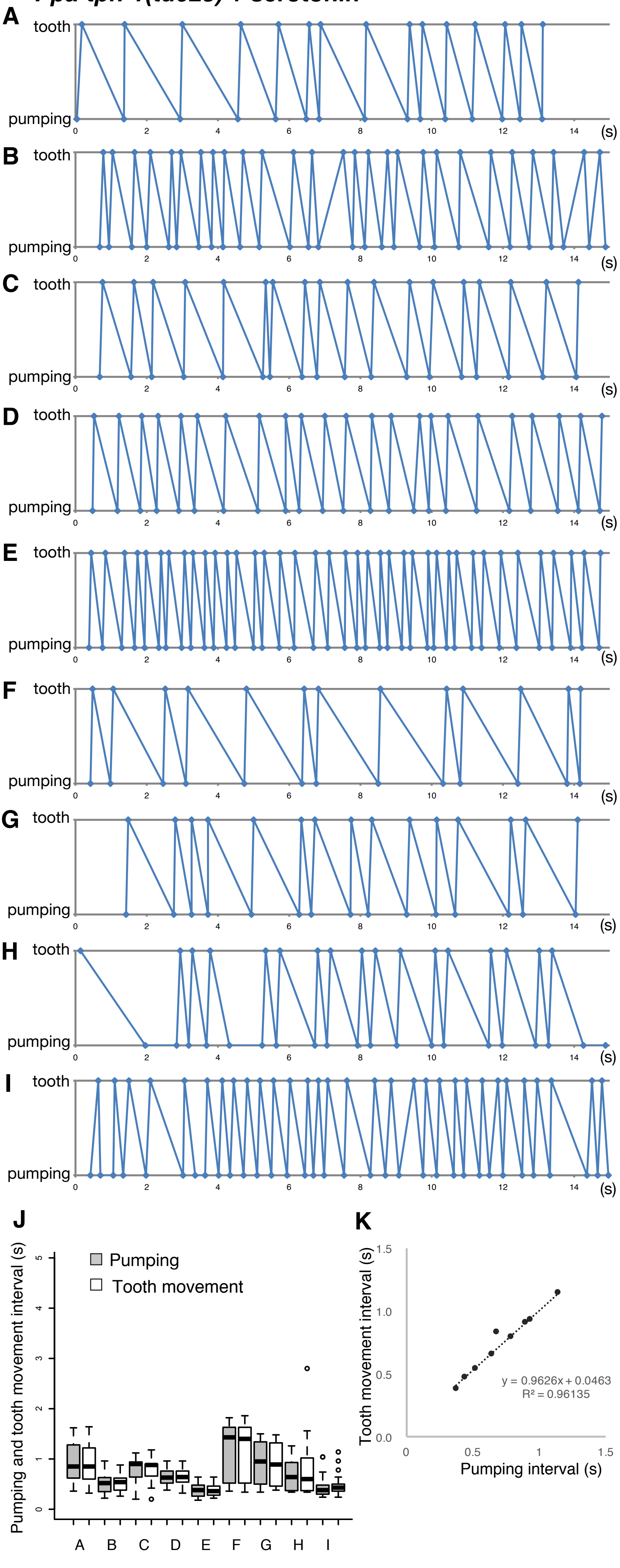

Figure S5

Ppa-tph-1(tu1016) + serotonin

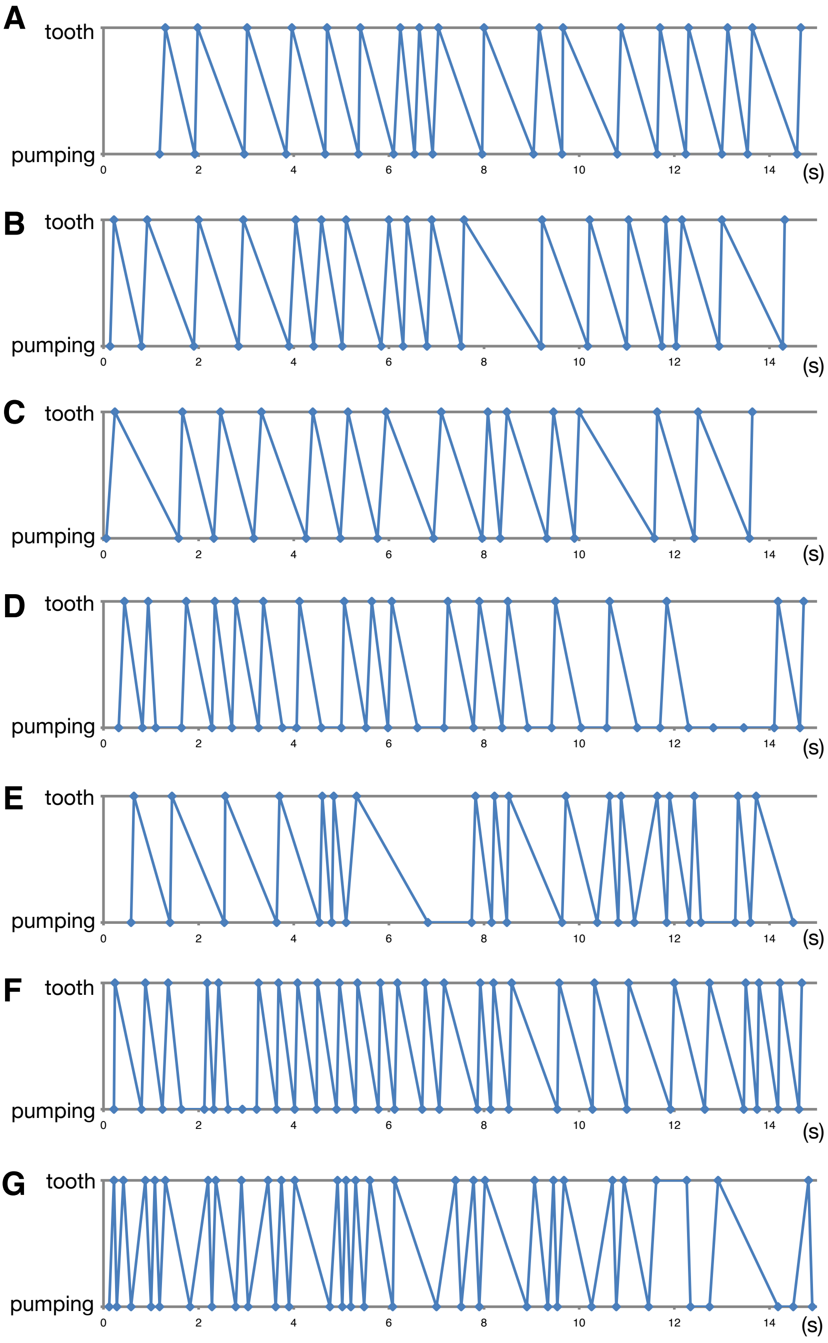

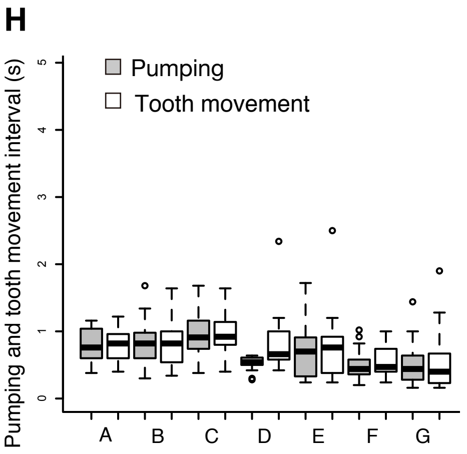

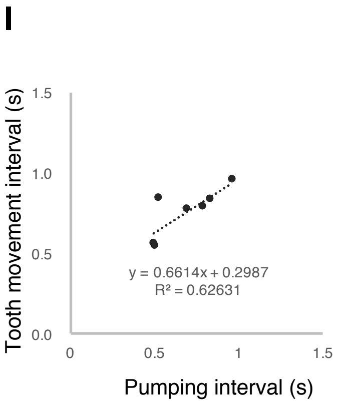

Figure S6

Ppa-bas-1(tu629)

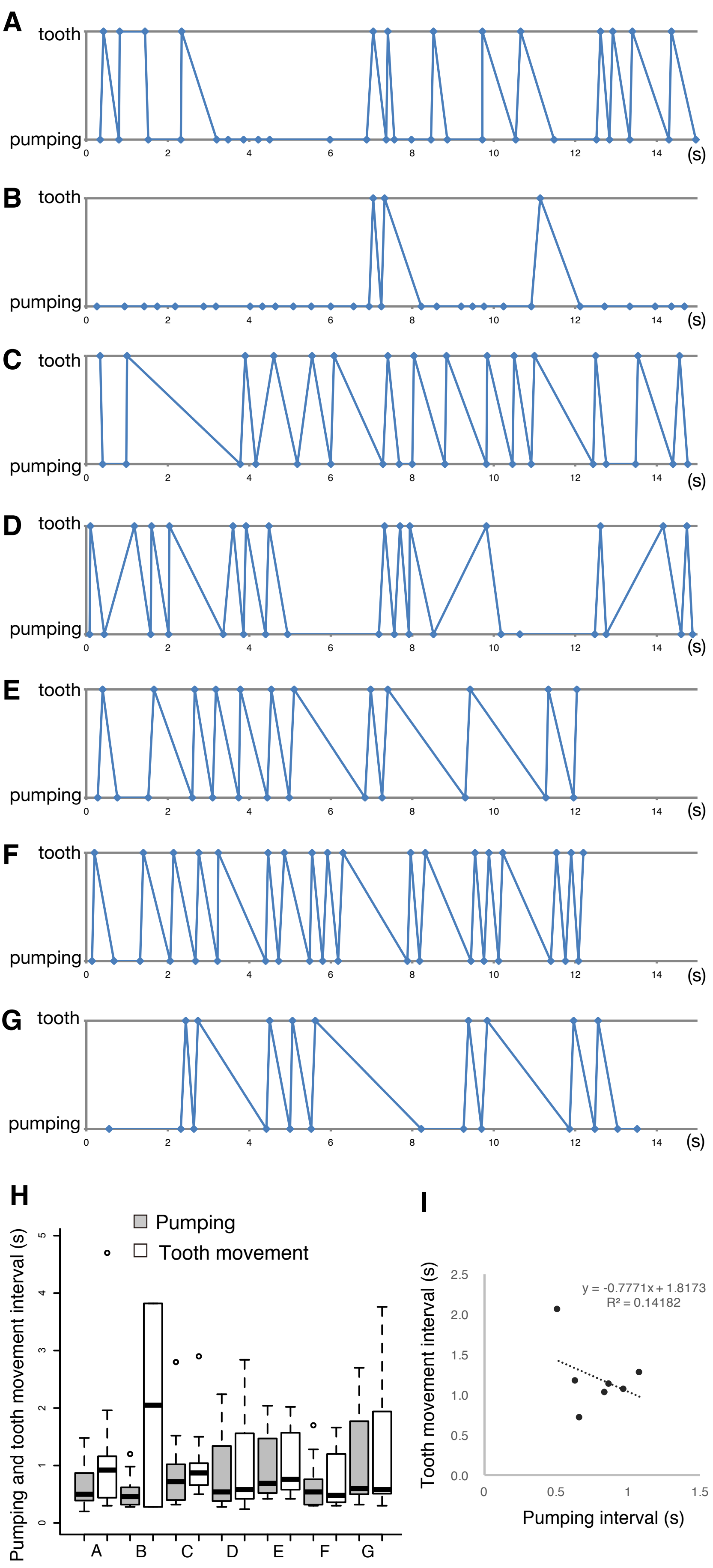

Figure S7

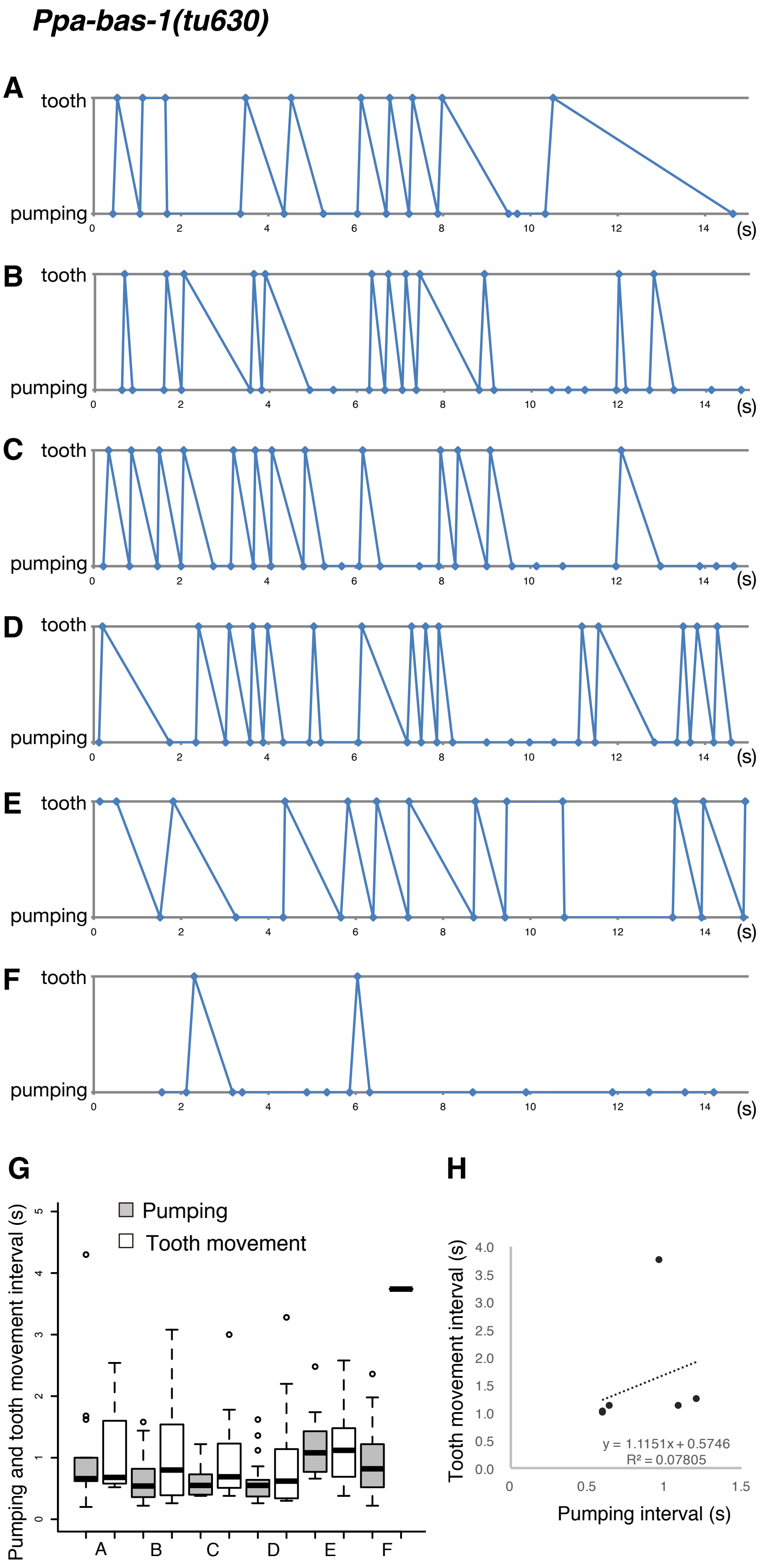

Ppa-bas-1(tu630)
A
B
C
D
E
F
tooth
pumping
0
2
4
6
8
10
12
14
(s)
G
Pumping
Tooth movement
Pumping and tooth movement interval (s)
H
Tooth movement interval (s)
Pumping interval (s)
y = 1.1151x + 0.5746
R² = 0.07805

Figure S8

Ppa-bas-1(tu629) + serotonin

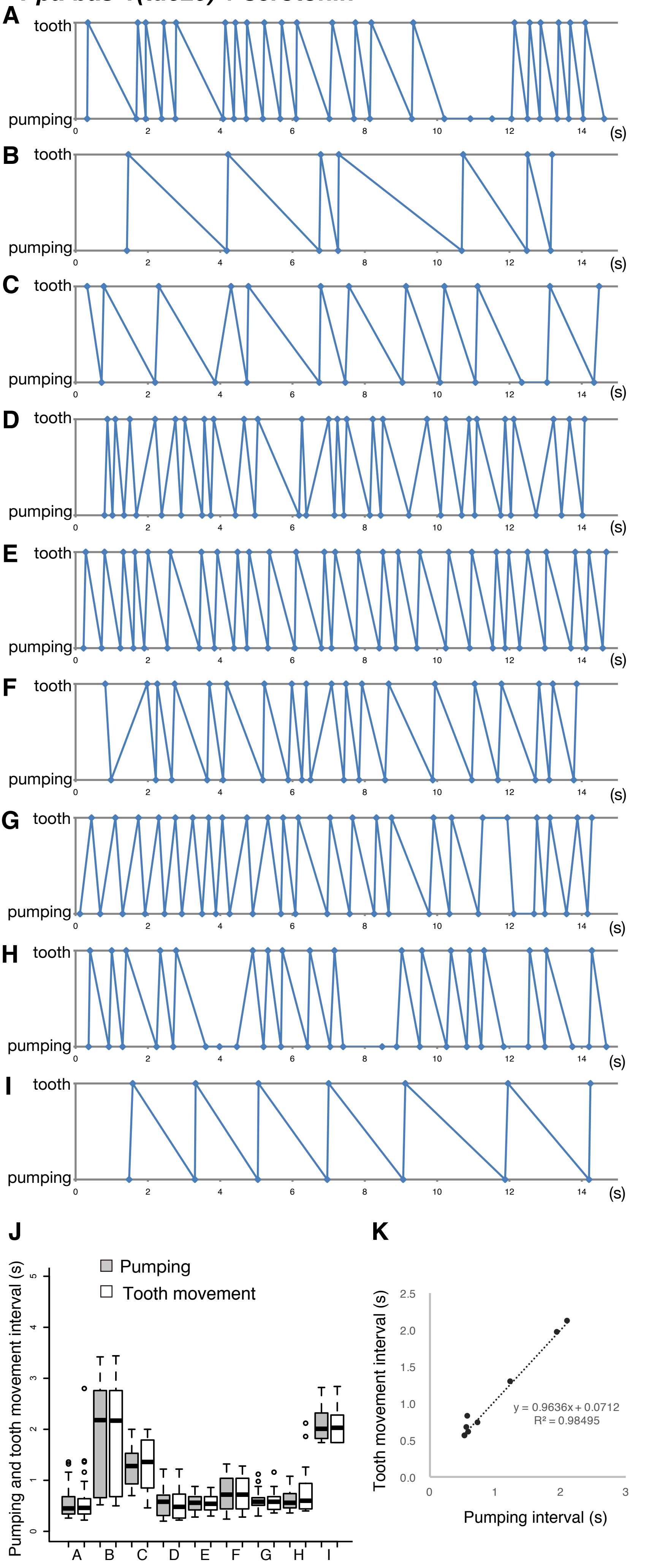

Figure S9

Ppa-tph-1(tu630) + serotonin

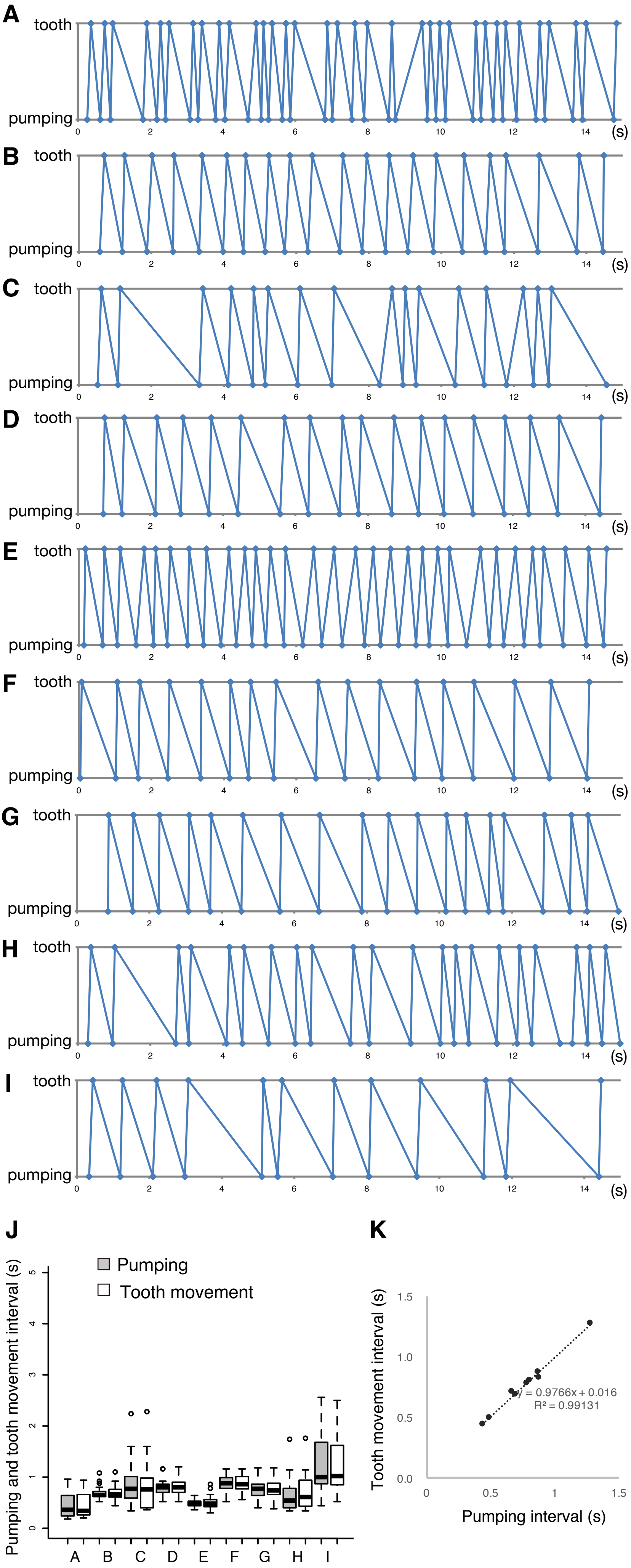

Figure S10

Ppa-bas-1(tu629) + dopamine

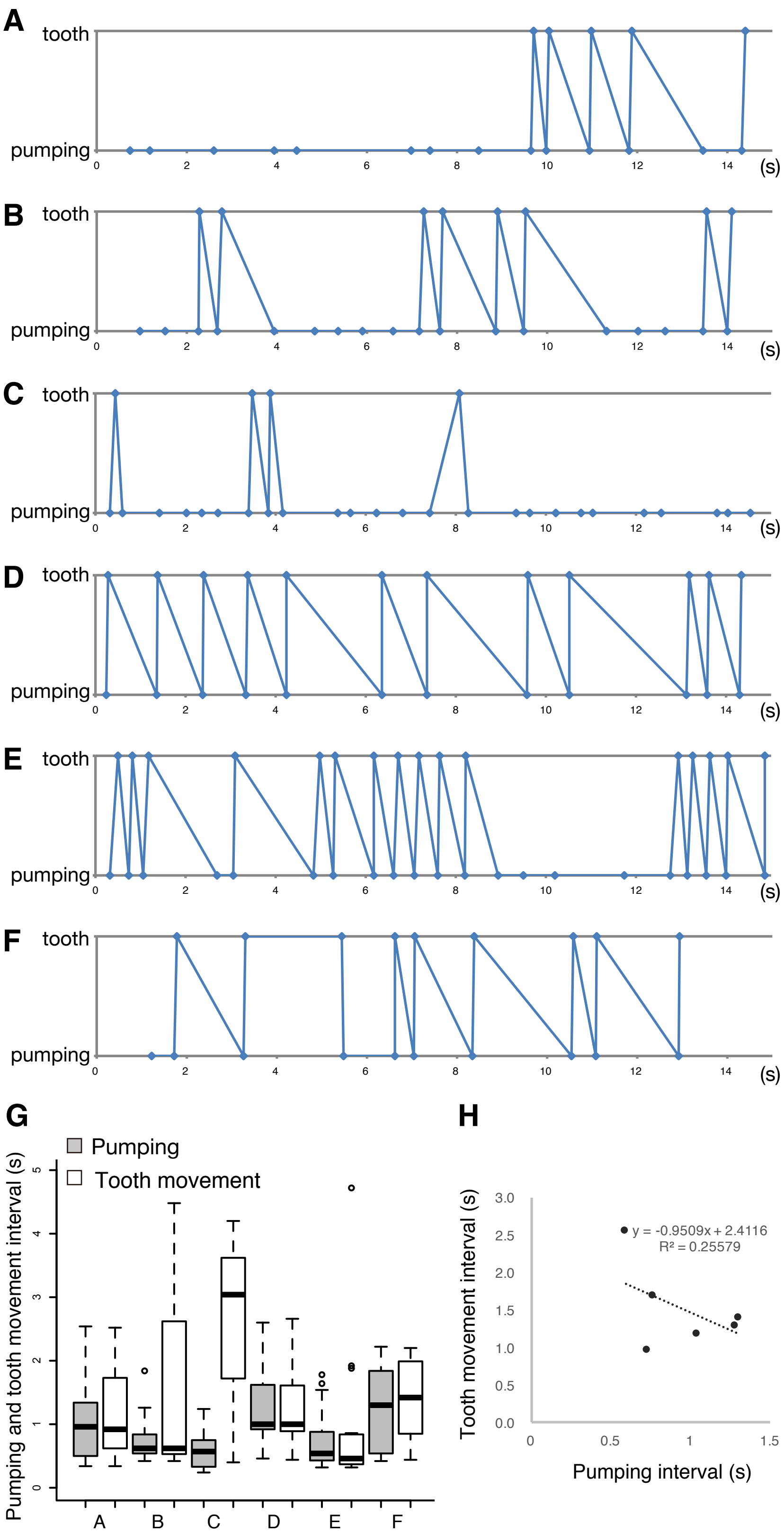

Figure S11

Ppa-bas-1(tu630) + dopamine

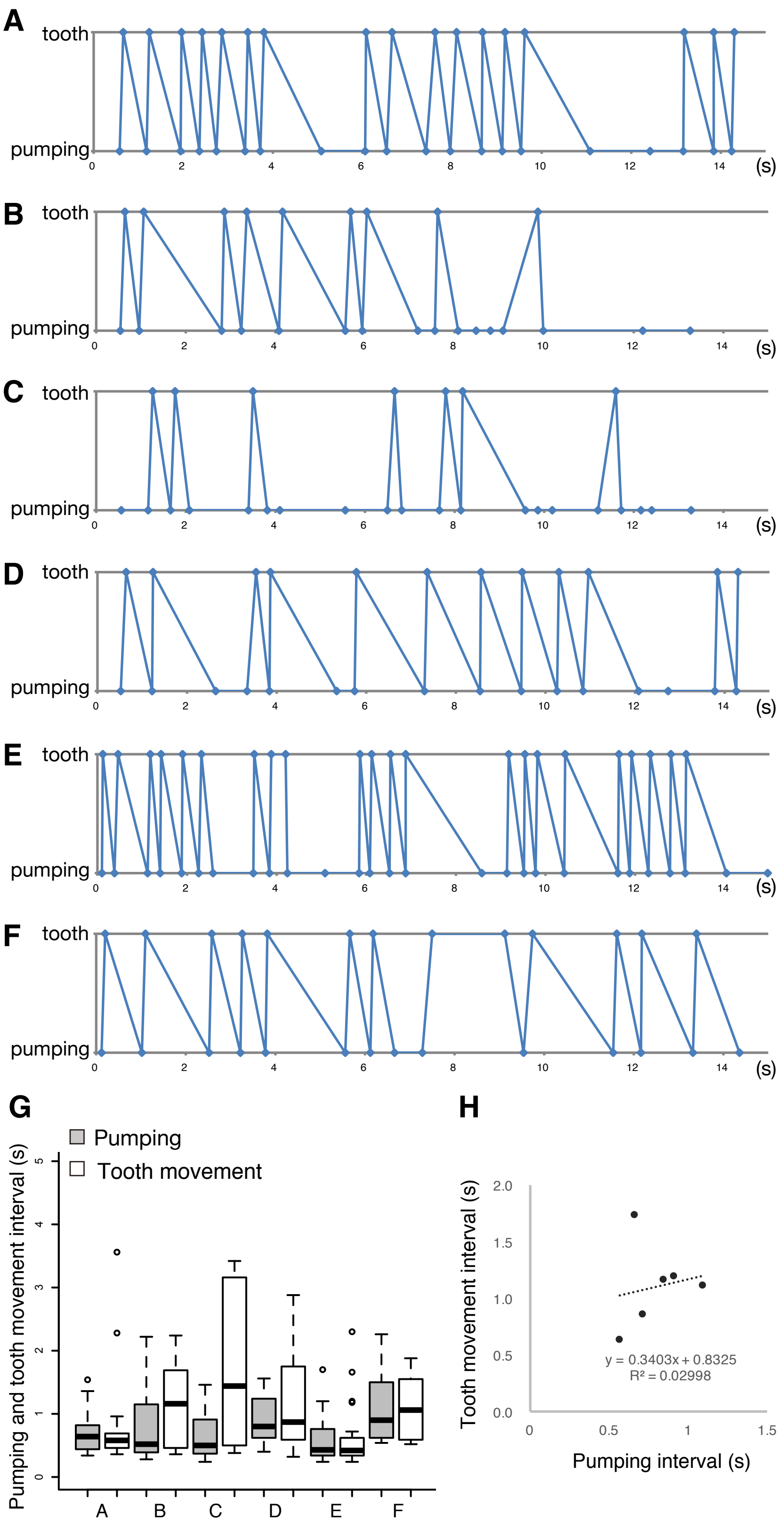

Figure S12

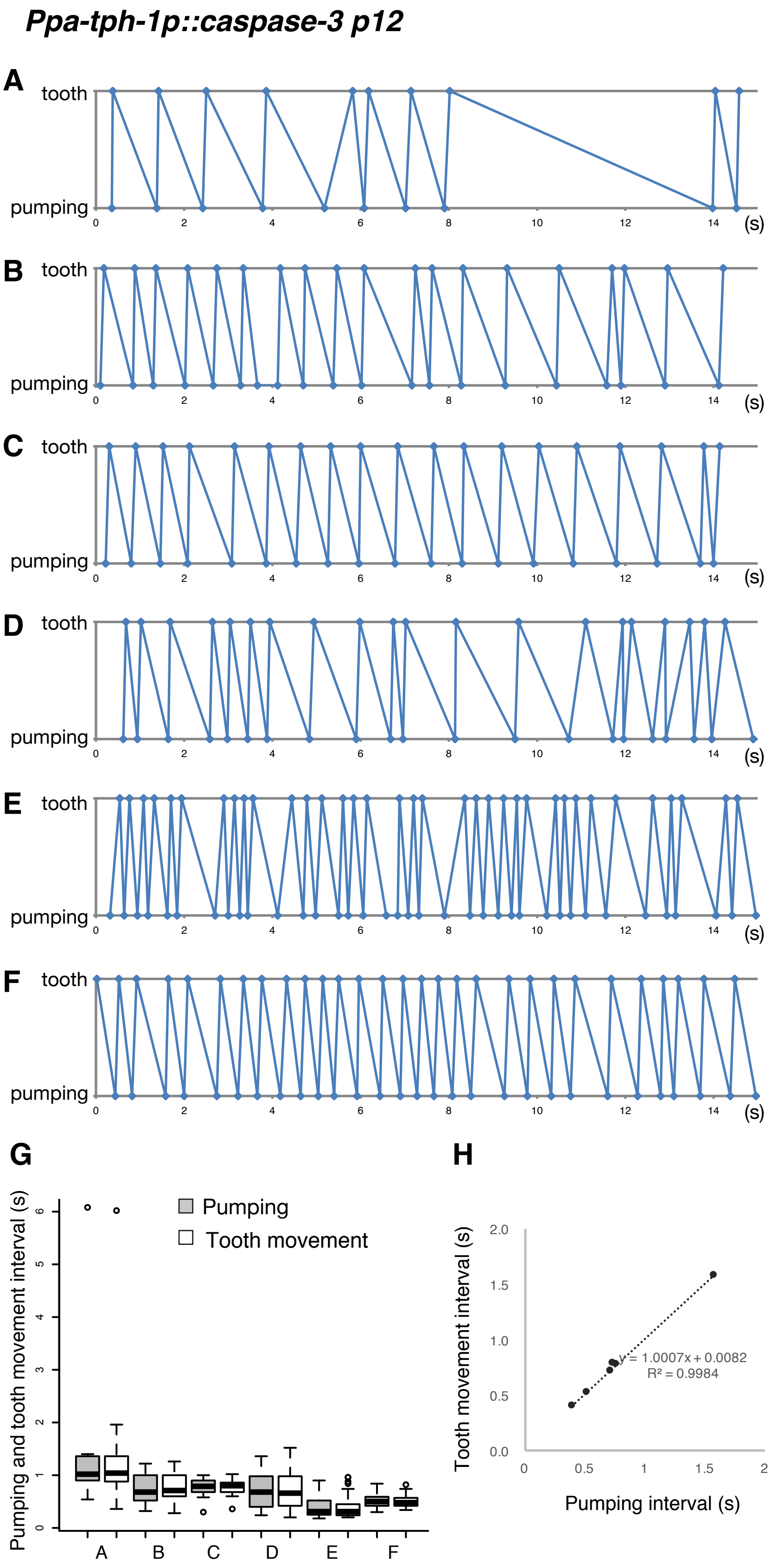

Ppa-tph-1p::caspase-3 p12
A
B
C
D
E
F
tooth
pumping
0
2
4
6
8
10
12
14
(s)
G
H
Pumping
Tooth movement
Pumping and tooth movement interval (s)
Tooth movement interval (s)
Pumping interval (s)
y = 1.0007x + 0.0082
R² = 0.9984

Figure S13

Ppa-tph-1p::caspase-3 p12, Ppa-tph-1p::caspase-3 p17

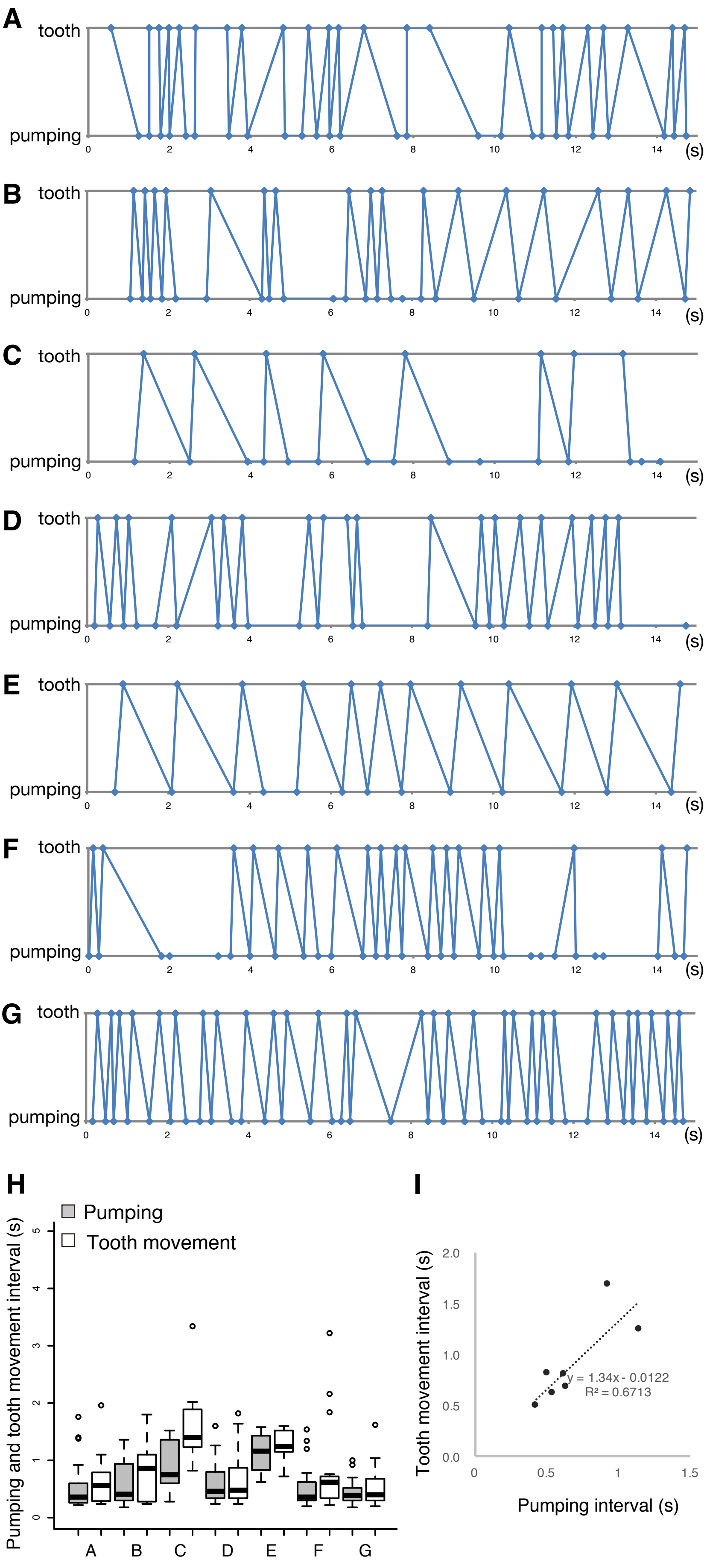

Figure S14

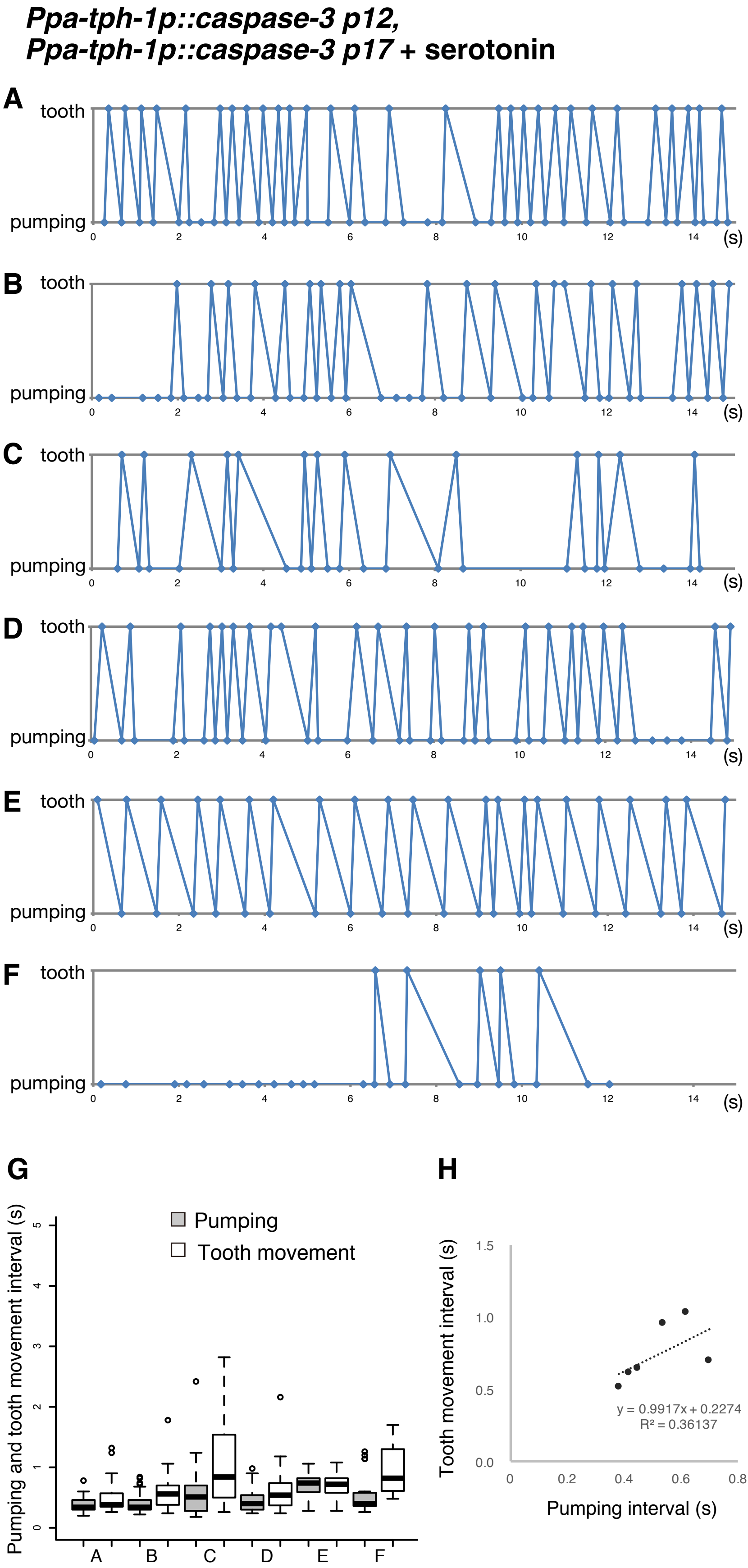

Ppa-tph-1p::caspase-3 p12,
Ppa-tph-1p::caspase-3 p17 + serotonin
A
B
C
D
E
F
tooth
pumping
0
2
4
6
8
10
12
14
(s)
G
Pumping
Tooth movement
Pumping and tooth movement interval (s)
A
B
C
D
E
F
H
Tooth movement interval (s)
Pumping interval (s)
y = 0.9917x + 0.2274
R² = 0.36137

Figure S1. Time plots of pumping and tooth movement in wild type.

A-F: Time plots of tooth closure (plotted as tooth) and muscle relaxation of corpus (plotted as pumping) in 15 seconds during predation. Each plot shows the result from different individuals. The timing of pumping and tooth movement was coordinated with a 1:1 ratio in wild type animals.

Figure S2. Time plots of pumping and tooth movement in *Ppa-tph-1(tu628)*.

A-J: Time plots of tooth closure (plotted as tooth) and muscle relaxation of corpus (plotted as pumping) in 15 seconds during predation. Each plot shows the result from different individuals. The timing coordination of pumping and tooth movement was disrupted in *Ppa-tph-1* mutants.

Figure S3. Time plots of pumping and tooth movement in *Ppa-tph-1(tu1016)*.

A-I: Time plots of tooth closure (plotted as tooth) and muscle relaxation of corpus (plotted as pumping) in 15 seconds during predation. Each plot shows the result from different individuals. J: Box plots show the individual variables of pumping intervals (grey) and tooth movement intervals (white) in *Ppa-tph-1(tu1016)* animals. Box and dark line within the box represent upper and lower quartiles and median, respectively. The whiskers (dashed lines) show the 1.5x quantile range and circles are outlier of this range. Items of the horizontal axis correspond to the individual results of Figure S3A–I. K: The relation between pumping intervals and tooth movement intervals of *Ppa-tph-1(tu1016)* animals was investigated using the Pearson's correlation coefficient test. The coordination of pumping and tooth movement was disrupted in *Ppa-tph-1* mutants.

Figure S4. Time plots of pumping and tooth movement in *Ppa-tph-1(tu628)* treated with serotonin.

A-I: Time plots of tooth closure (plotted as tooth) and muscle relaxation of corpus (plotted as pumping) in 15 seconds during predation. Each plot shows the result from different individuals. J: Box plots show the individual variables of the pumping interval (grey) and the tooth movement interval (white) in *Ppa-tph-1(tu628)* animals treated with serotonin. Box and dark line within the box represent upper and lower quartiles and median, respectively. The whiskers (dashed lines) show the 1.5x quantile range and circles are outlier of this range. Items of the horizontal axis correspond to the individual results of

Figure S4A–I. K: The relation between pumping intervals and tooth movement intervals of *Ppa-tph-1(tu628)* animals treated with serotonin was investigated using the Pearson's correlation coefficient test. Treatment with serotonin before the assay restored the uncoordinated defect in *Ppa-tph-1* mutants.

Figure S5. Time plots of pumping and tooth movement in *Ppa-tph-1(tu1016)* treated with serotonin.

A-G: Time plots of tooth closure (plotted as tooth) and muscle relaxation of corpus (plotted as pumping) in 15 seconds during predation. Each plot shows the result from different individuals. H: Box plots show the individual variables of pumping intervals (grey) and tooth movement intervals (white) in *Ppa-tph-1(tu628)* animals treated with serotonin. Box and dark line within the box represent upper and lower quartiles and median, respectively. The whiskers (dashed lines) show the 1.5x quantile range and circles are outlier of this range. Items of the horizontal axis correspond to the individual results of Figure S5A–G. I: The relation between pumping intervals and tooth movement intervals of *Ppa-tph-1(tu1016)* animals treated with serotonin was investigated using the Pearson's correlation coefficient test. Treatment with serotonin before the assay restored the uncoordinated defect in *Ppa-tph-1* mutants.

Figure S6. Time plots of pumping and tooth movement in *Ppa-bas-1(tu629)*.

A-G: Time plots of tooth closure (plotted as tooth) and muscle relaxation of corpus (plotted as pumping) in 15 seconds during predation. Each plot shows the result from different individuals. H: Box plots show the individual variables of the pumping interval (grey) and the tooth movement interval (white) in *Ppa-bas-1(tu629)* animals. Box and dark line within the box represent upper and lower quartiles and median, respectively. The whiskers (dashed lines) show the 1.5x quantile range and circles are outlier of this range. Items of the horizontal axis correspond to the individual results of Figure S6A–G. I: The relation between pumping intervals and tooth movement intervals of *Ppa-bas-1(tu629)* animals was investigated using the Pearson's correlation coefficient test. The coordination of pumping and tooth movement was disrupted in *Ppa-bas-1* mutants.

Figure S7. Time plots of pumping and tooth movement in *Ppa-bas-1(tu630)*.

A-F: Time plots of tooth closure (plotted as tooth) and muscle relaxation of corpus

(plotted as pumping) in 15 seconds during predation. Each plot shows the result from different individuals. G: Box plots show the individual variables of the pumping interval (grey) and the tooth movement interval (white) in *Ppa-bas-1(tu630)* animals. Box and dark line within the box represent upper and lower quartiles and median, respectively. The whiskers (dashed lines) show the 1.5x quantile range and circles are outlier of this range. Items of the horizontal axis correspond to the individual results of Figure S7A–F. H: The relation between pumping intervals and tooth movement intervals of *Ppa-bas-1(tu630)* animals was investigated using the Pearson's correlation coefficient test. The coordination of pumping and tooth movement was disrupted in *Ppa-bas-1* mutants.

Figure S8. Time plots of pumping and tooth movement in *Ppa-bas-1(tu629)* treated with serotonin.

A-I: Time plots of tooth closure (plotted as tooth) and muscle relaxation of corpus (plotted as pumping) in 15 seconds during predation. Each plot shows the result from different individuals. J: Box plots show the individual variables of the pumping interval (grey) and the tooth movement interval (white) in *Ppa-bas-1(tu629)* animals treated with serotonin. Box and dark line within the box represent upper and lower quartiles and median, respectively. The whiskers (dashed lines) show the 1.5x quantile range and circles are outlier of this range. Items of the horizontal axis correspond to the individual results of Figure S8A–I. K: The relation between pumping intervals and tooth movement intervals of *Ppa-bas-1(tu629)* treated with serotonin was investigated using the Pearson's correlation coefficient test. Treatment with serotonin before the assay restored the uncoordinated defect in *Ppa-bas-1* mutants.

Figure S9. Time plots of pumping and tooth movement in *Ppa-bas-1(tu630)* treated with serotonin.

A-I: Time plots of tooth closure (plotted as tooth) and muscle relaxation of corpus (plotted as pumping) in 15 seconds during predation. Each plot shows the result from different individuals. J: Box plots show the individual variables of the pumping interval (grey) and the tooth movement interval (white) in *Ppa-bas-1(tu630)* animals treated with serotonin. Box and dark line within the box represent upper and lower quartiles and median, respectively. The whiskers (dashed lines) show the 1.5x quantile range and circles are outlier of this range. Items of the horizontal axis correspond to the individual results of

Figure S9A–I. K: The relation between pumping intervals and tooth movement intervals of *Ppa-bas-1(tu630)* treated with serotonin was investigated using the Pearson's correlation coefficient test. Treatment with serotonin before the assay restored the uncoordinated defect in *Ppa-bas-1* mutants.

Figure S10. Time plots of pumping and tooth movement in *Ppa-bas-1(tu629)* treated with dopamine.

A-F: Time plots of tooth closure (plotted as tooth) and muscle relaxation of corpus (plotted as pumping) in 15 seconds during predation. Each plot shows the result from different individuals. G: Box plots show the individual variables of the pumping interval (grey) and the tooth movement interval (white) in *Ppa-bas-1(tu629)* animals treated with dopamine. Box and dark line within the box represent upper and lower quartiles and median, respectively. The whiskers (dashed lines) show the 1.5x quantile range and circles are outlier of this range. Items of the horizontal axis correspond to the individual results of Figure S10A–F. H: The relation between pumping intervals and tooth movement intervals of *Ppa-bas-1(tu629)* treated with dopamine was investigated using the Pearson's correlation coefficient test. Treatment with dopamine before the assay did not restored the uncoordinated defect in *Ppa-bas-1* mutants.

Figure S11. Time plots of pumping and tooth movement in *Ppa-bas-1(tu630)* treated with dopamine.

A-F: Time plots of tooth closure (plotted as tooth) and muscle relaxation of corpus (plotted as pumping) in 15 seconds during predation. Each plot shows the result from different individuals. G: Box plots show the individual variables of the pumping interval (grey) and the tooth movement interval (white) in *Ppa-bas-1(tu630)* animals treated with dopamine. Box and dark line within the box represent upper and lower quartiles and median, respectively. The whiskers (dashed lines) show the 1.5x quantile range and circles are outlier of this range. Items of the horizontal axis correspond to the individual results of Figure S11A–F. H: The relation between pumping intervals and tooth movement intervals of *Ppa-bas-1(tu630)* treated with dopamine was investigated using the Pearson's correlation coefficient test. Treatment with dopamine before the assay did not restored the uncoordinated defect in *Ppa-bas-1* mutants.

Figure S12. Time plots of pumping and tooth movement in control animals.

A-F: Time plots of tooth closure (plotted as tooth) and muscle relaxation of corpus (plotted as pumping) in 15 seconds during predation. Each plot shows the result from different individuals. G: Box plots show the individual variables of the pumping interval (grey) and the tooth movement interval (white) in control animals. Box and dark line within the box represent upper and lower quartiles and median, respectively. The whiskers (dashed lines) show the 1.5x quantile range and circles are outlier of this range. Items of the horizontal axis correspond to the individual results of Figure S12A–F. H: The relation between pumping intervals and tooth movement intervals of control animals was investigated using the Pearson's correlation coefficient test. The timing of pumping and tooth movement was coordinated with a 1:1 ratio in control animals that express only a single subunit of the reconstituted caspases.

Figure S13. Time plots of pumping and tooth movement in NSM and ADF ablated worms.

A-G: Time plots of tooth closure (plotted as tooth) and muscle relaxation of corpus (plotted as pumping) in 15 seconds during predation. Each plot shows the result from different individuals. H: Box plots show the individual variables of the pumping interval (grey) and the tooth movement interval (white) in ablated animals. Box and dark line within the box represent upper and lower quartiles and median, respectively. The whiskers (dashed lines) show the 1.5x quantile range and circles are outlier of this range. Items of the horizontal axis correspond to the individual results of Figure S13A–G. I: The relation between pumping intervals and tooth movement intervals of ablated animals was investigated using the Pearson's correlation coefficient test. The coordination of pumping and tooth movement was disrupted in the animals that express both subunits of reconstituted caspases in NSM and ADF.

Figure S14. Time plots of pumping and tooth movement in NSM and ADF ablated worms treated with serotonin.

A-F: Time plots of tooth closure (plotted as tooth) and muscle relaxation of corpus (plotted as pumping) in 15 seconds during predation. Each plot shows the result from different individuals. G: Box plots show the individual variables of the pumping interval (grey) and the tooth movement interval (white) in ablated animals treated with serotonin.

Box and dark line within the box represent upper and lower quartiles and median, respectively. The whiskers (dashed lines) show the 1.5x quantile range and circles are outlier of this range. Items of the horizontal axis correspond to the individual results of Figure S14A–F. H: The relation between pumping intervals and tooth movement intervals of ablated animals treated with serotonin was investigated using the Pearson's correlation coefficient test. Treatment with serotonin before the assay did not restored the uncoordinated defect in NSM and ADF ablated worms.
